# Aryl Hydrocarbon Receptor Promotes IL-10 Expression in Inflammatory Macrophages Through Src-STAT3 Signaling Pathway

**DOI:** 10.3389/fimmu.2018.02033

**Published:** 2018-09-19

**Authors:** Junyu Zhu, Li Luo, Lixing Tian, Shangqi Yin, Xiaoyuan Ma, Shaowen Cheng, Wanqi Tang, Jing Yu, Wei Ma, Xiaoying Zhou, Xia Fan, Xue Yang, Jun Yan, Xiang Xu, Chuanzhu Lv, Huaping Liang

**Affiliations:** ^1^State Key Laboratory of Trauma, Burns and Combined Injury, Research Institute of Surgery, Daping Hospital, Army Medical University, Chongqing, China; ^2^Emergency and Trauma College of Hainan Medical University, Second Affiliated Hospital of Hainan Medical University, Haikou, China; ^3^Trauma Center, First Affiliated Hospital of Hainan Medical University, Haikou, China

**Keywords:** aryl hydrocarbon receptor, IL-10, macrophages, inflammation, signal transduction

## Abstract

The aryl hydrocarbon receptor (AhR) is an important immune regulator with a role in inflammatory response. However, the role of AhR in IL-10 production by inflammatory macrophages is currently unknown. In this study, we investigated LPS-induced IL-10 expression in macrophages from AhR-KO mice and AhR-overexpressing RAW264.7 cells. AhR was highly expressed after LPS stimulation through NF-κB pathway. Loss of AhR resulted in reduced IL-10 expression in LPS-induced macrophages. Moreover, the IL-10 expression was elevated in LPS-induced AhR-overexpressing RAW264.7 cells. Maximal IL-10 expression was dependent on an AhR non-genomic pathway closely related to Src and STAT3. Furthermore, AhR-associated Src activity was responsible for tyrosine phosphorylation of STAT3 and IL-10 expression by inflammatory macrophages. Adoptive transfer of AhR-expressing macrophages protected mice against LPS-induced peritonitis associated with high IL-10 production. In conclusion, we identified the AhR-Src-STAT3-IL-10 signaling pathway as a critical pathway in the immune regulation of inflammatory macrophages, It suggests that AhR may be a potential therapeutic target in immune response.

## Introduction

Inflammation is a double-edged sword of the innate immune response. To limit the undesirable consequences of excessive inflammation, factors that modulate the initiation phase and the resolution phase of inflammation can determine the nature of the inflammatory response (1). The aryl hydrocarbon receptor (AhR) is a ligand-activated transcription factor that was initially recognized as a receptor mediating the pathologic effects of dioxins and other pollutants (2). Recent studies have identified the molecular functions of AhR in the immune system during steady state and during infection and inflammation (3–6). AhR was shown to be involved in LPS-induced inflammatory gene expression (7). AhR-KO mice were hypersensitive to LPS-induced septic shock, mainly as a result of macrophage dysfunction. Consistent with their enhanced susceptibility to LPS treatment, AhR-KO mice showed markedly increased plasma levels of IL-1β, IL-18, IL-6, and

TNF-α (8). Activated AhR also played a central role in limiting endotoxin-triggered inflammation, resulting in the establishment of endotoxin tolerance (9).

Inflammatory responses mediated by macrophages are part of the innate immune system (10). Macrophages are important effector cells of innate immunity, with a pivotal role in host defense against intracellular pathogens (11). The roles of AhR in the differentiation and function of specific T-cell subpopulations and B cells in adaptive immune response are well known (4). However, although numerous studies have addressed the modulatory effect of AhR in innate immune cells, such as dendritic cells (DCs), neutrophils and natural killer cells (12), the exact role of AhR in macrophage function remains to be elucidated. Increasing evidences have also demonstrated roles for AhR in the regulation of inflammation and inflammatory cytokines. AhR negatively regulated IL-6 production in macrophages following LPS stimulation (13, 14). AhR activation also inhibited caspase-1 activation and subsequent IL-1β secretion in macrophages (15). IL-10 is an immunoregulatory cytokine with a crucial role in ameliorating immunopathology and preventing inflammatory responses, which leads us to hypothesize that AhR may also regulate IL-10 expression in inflammatory macrophages in innate immunity.

IL-10 is an important anti-inflammatory cytokine, and understanding how its expression is critical to designing immune-intervention strategies (16). IL-10 production is regulated by various transcription factors and signaling pathways. Src kinases are a family of nine non-receptor tyrosine kinases involved in the induction of IL-10 (17, 18). Acute alcohol exposure activates many transcription factors via the Src kinase family to promote IL-10 production in human monocytes (19). STAT3 is an important regulator of LPS-induced IL-10 gene expression (20) and has been shown to bind to intron 4 of the IL-10 gene and drive its production (21). Notably, STAT3 activation also occurs downstream of the IL-10 receptor (22), and IL-10 positively regulates its own production in an autocrine manner via activation of STAT3 (23). It is therefore necessary to determine if feed-forward IL-10 loop exists in the study. Furthermore, several studies have shown that activation of MAPK (24, 25) and the PI(3)K-AKT-mTOR pathways (26) are critical for the production of IL-10 by macrophages.

Previous reports revealed that AhR function was mediated by both genomic and non-genomic pathways. The AhR canonical pathway that is also called as AhR genomic pathway is characterized by AhR nuclear translocation. In the absence of a ligand, AhR is present in the cytoplasm in a complex with several chaperone proteins, including heat shock protein 90 (HSP90), p23, X-associated protein 2, and AhR associated protein 9. Upon ligand binding, AhR translocates into the nucleus, where it is released from the complex, heterodimerizes with its protein partner AhR nuclear translocator (ARNT), and finally binds to genomic regions inducing transcription of its target genes (3, 27). However, in addition to this classical AhR genomic pathway, the AhR also has been shown to regulate gene expression through non-genomic signaling pathways (4, 28). Several studies reported that 2,3,7,8-tetrachlorodibenzo p-dioxin (TCDD) induced inflammatory responses through a non-genomic AhR function (29, 30). In the absence of ligand activation, AhR could control cellular processes along with other transcriptional factors in the cytoplasm (9, 13, 28). It is therefore necessary to determine if the above pathways are involved in the regulation of IL-10 expression by AhR.

In this study, we investigated LPS-induced AhR expression in various macrophages and its role in IL-10 expression. We examined the mechanisms responsible for LPS-stimulated IL-10 production in macrophages from AhR-KO mice and AhR-overexpressing RAW264.7 cells, and showed that it was associated with Src and STAT3 activation, but was not regulated via the AhR genomic pathway. These results identified the AhR-Src-STAT3 pathway as a critical signaling pathway in the production of IL-10 by inflammatory macrophages, thus providing an innovative platform for future studies of the mechanisms of AhR in immune regulation.

## Results

### AhR is increased in LPS-stimulated macrophages via NF-κB pathway

**Figure 1:**
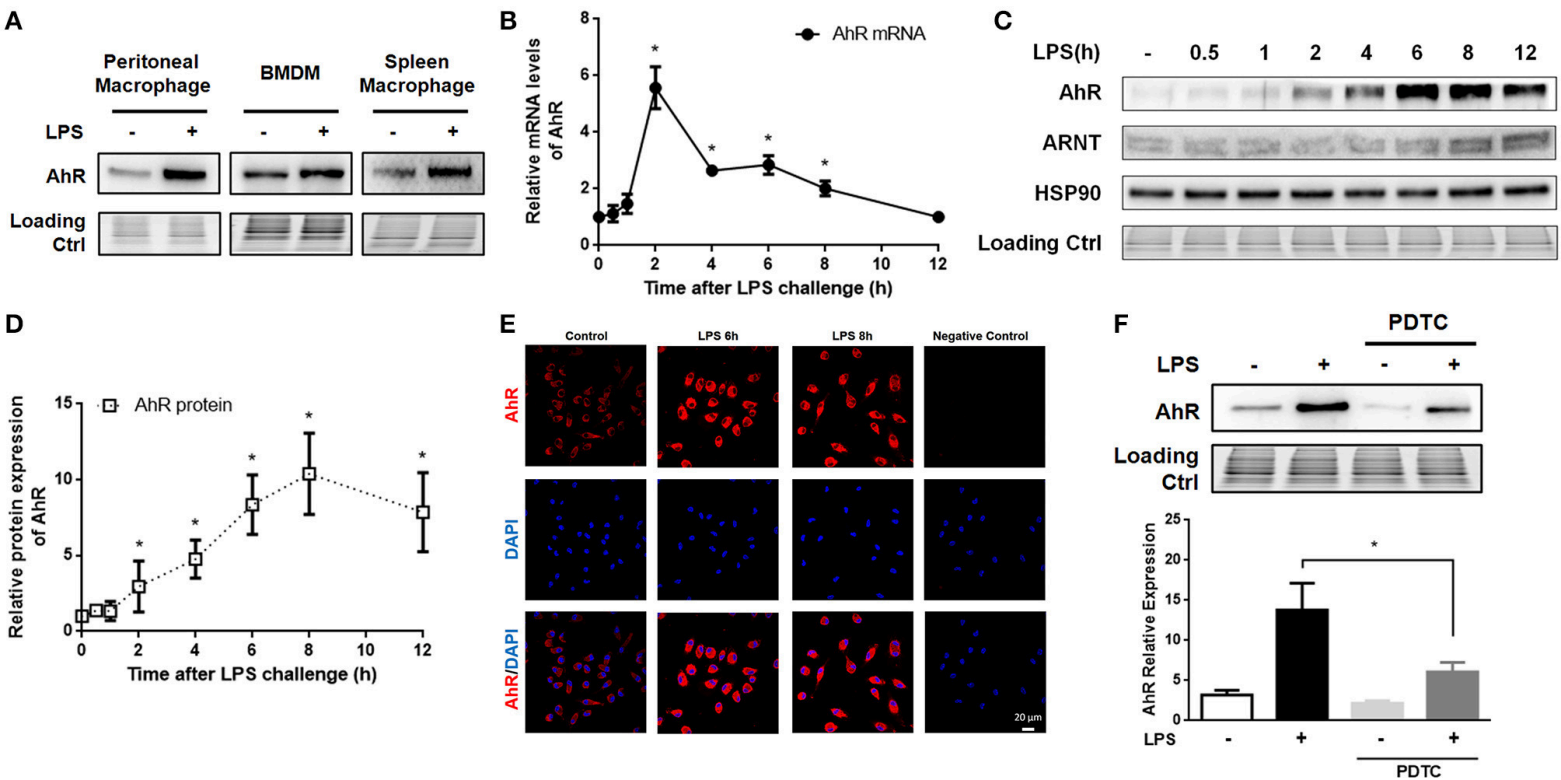
AhR was increased in LPS-stimulated macrophages via the NF-κB pathway. **(A)** Peritoneal macrophages, BMDMs, and spleen macrophages isolated from C57BL/6J mice were treated with or without LPS (10 μg/ml) for 8 h. Then, the cells were lysed and subjected to western blotting analysis of AhR protein expression. **(B–E)** Peritoneal macrophages were treated with or without LPS (10 μg/ml) for the indicated times. **(B)** AhR mRNA levels were detected with qRT-PCR. The experiment was repeated three times; combined results are compared using one-way ANOVA test (2 h: control, *P* < 0.001; 4 h, control; *P* < 0.001; 6 h, control; *P* < 0.001; 8 h, control; *P* = 0.004). **(C)** Protein expression levels of AhR, ARNT, and HSP90 were analyzed with western blotting. **(D)** Quantitative analysis of the AhR expression measured in **(C)**. The experiment was repeated four times; combined results are compared using one-way ANOVA test (2 h: control, *P* < 0.001; 4 h: control, *P* < 0.001; 6 h: control, *P* < 0.001; 8 h: control, *P* = 0.004). **(E)** AhR protein expression was also assessed with immunofluorescence. Cells were immunostained with AhR-specific antibody (red) and co-stained with DAPI (blue) to detect nuclei. Bar, 20 μm. **(F)** Peritoneal macrophages were pre-treated with PDTC for 1 h and then stimulated with LPS for 8 h. AhR protein expression was analyzed by western blotting. The experiment was repeated three times; combined results are compared using one-way ANOVA test (LPS: LPS+PDTC, *P* = 0.013). Western blots and immunofluorescence images are representative of three independent experiments. Data shown as mean ± SEM of at least three independent experiments. Differences were analyzed with one-way ANOVA. ^*^P < 0.05.

AhR was previously reported to be highly expressed in T cells stimulated by TGF-β plus IL-6 (31) and in LPS-induced bone marrow derived DCs (32) and human DCs (33). In this study, we investigated the expression of AhR in LPS-stimulated mouse macrophages, which were identified according to the dual presence of F4/80 and CD11b (Figure S1). LPS increased AhR expression in peritoneal macrophages, bone marrow derived macrophages (BMDMs) and spleen macrophages (Figure 1A). AhR mRNA levels of peritoneal macrophages were increased at 1 h after LPS treatment, reached a peak at 2 h, and then gradually decreased (Figure 1B). Consistent with the mRNA data, AhR protein expression levels were increased at 2 h after LPS stimulation, increased rapidly up to 12 h, and peaked at 8 h (Figures 1C,D). However, LPS had no significant effect on expression of the AhR chaperone proteins ARNT and HSP90 (Figure 1C). Immunofluorescence assay confirmed that AhR protein expression was enhanced at 6 and 8 h after LPS stimulation (Figure 1E). Given that activated NF-κB is a critical component regulating the expression of AhR (33), we examined AhR expression in the presence of the NF-κB inhibitor pyrrolidinedithiocarbamate (PDTC). Peritoneal macrophages were pretreated with PDTC for 1 h, and the inhibitory effect was confirmed by reduced translocation of p65 and p50 into the nucleus after NF-κB activation. The LPS-stimulated AhR expression was disturbed by pretreatment with PDTC (Figure 1F). These results supported the hypothesis that LPS-induced AhR expression was mediated by the NF-κB pathway in macrophages.

### Loss of AhR in macrophages results in reduced IL-10 expression after LPS stimulation

**Figure 2:**
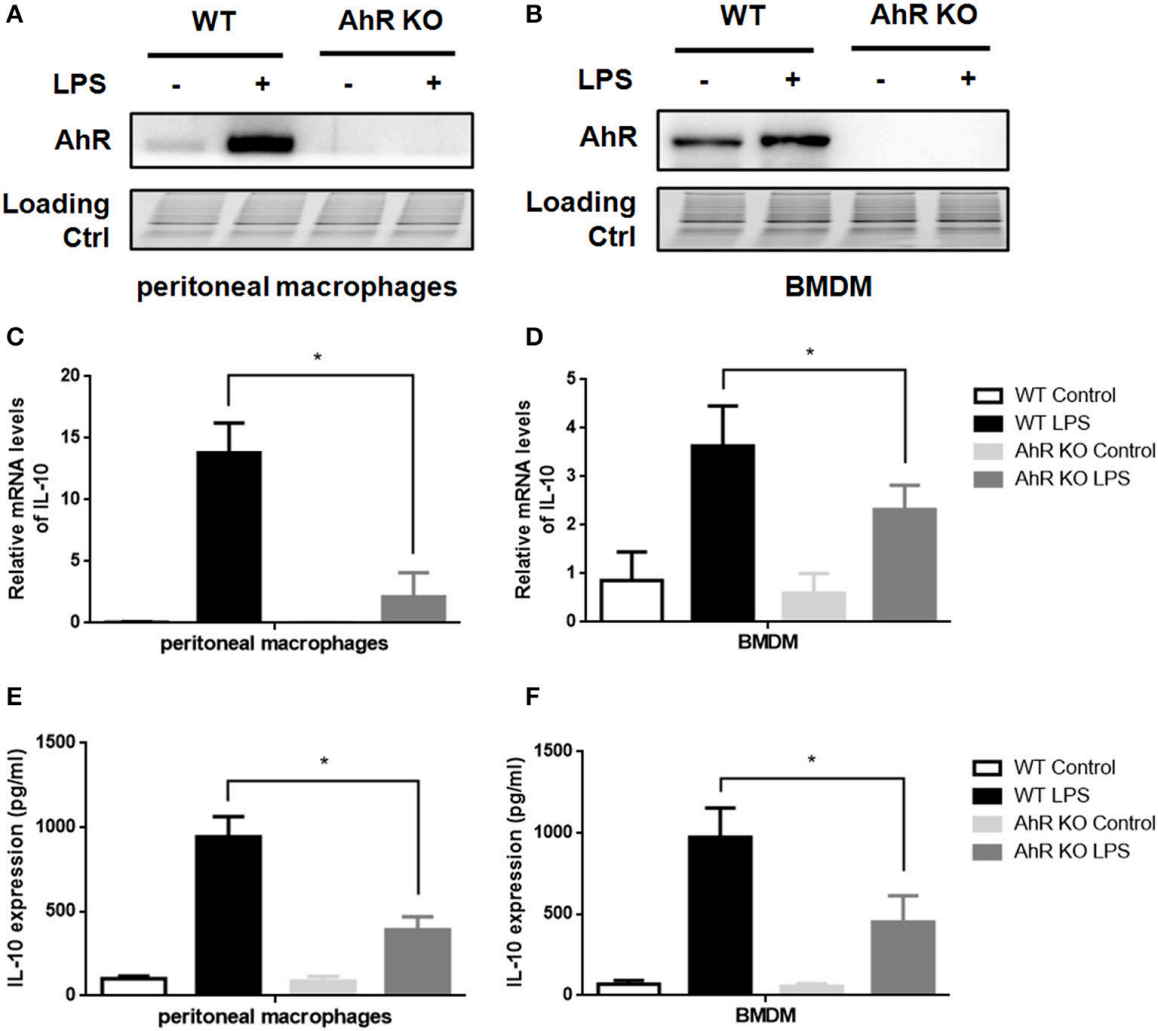
Loss of AhR in macrophages reduced IL-10 expression after LPS stimulation. Peritoneal macrophages and BMDMs isolated from WT or AhR-KO mice were stimulated with or without LPS at the indicated time points. **(A,B)** Whole-cell lysates were subjected to western blotting analysis with anti-AhR antibody. **(C,D)** IL-10 mRNA levels were detected with qRT-PCR at 4 h after LPS stimulation. The experiment was repeated three times; combined results are compared using one-way ANOVA test (PMs, *P* < 0.001; BMDMs, *P* < 0.001). **(E,F)** Supernatants were collected at 12 h after LPS stimulation and IL-10 production was measured with ELISA. The experiment was repeated six times; combined results are compared using one-way ANOVA test (PMs, *P* < 0.001; BMDMs, *P* = 0.003). Western blots images are representative of three independent experiments. Data in all bar graphs are mean ± SEM of three independent experiments. Differences were analyzed by one-way ANOVA. ^*^P < 0.05.

AhR downregulated IL-6 (13, 14) and IL-1β (15) expression in inflammatory macrophages in line with our current findings (Figure S2). Because AhR acted as a negative regulator of TLR signaling pathway, we speculated that AhR was positively correlated with IL-10 expression in LPS-induced macrophages. AhR expression levels in AhR-KO cells were greatly reduced compared with WT cells (Figures 2A,B). We also compared LPS-induced IL-10 expression in WT and AhR-KO macrophages, and IL-10 mRNA levels were reduced in peritoneal macrophages from AhR-KO mice compared with WT mice at 4 h after LPS stimulation (Figure 2C). Levels of IL-10 protein in the culture medium of LPS-induced AhR-KO peritoneal macrophages were lower than that of LPS-induced WT cells (Figure 2E). Furthermore, BMDMs from AhR-KO mice produced less IL-10 mRNA (Figure 2D) and IL-10 protein (Figure 2F) in response to LPS compared with BMDMs from WT mice. These results suggested that AhR expression was positively correlated with IL-10 expression in inflammatory macrophages.

### AhR upregulated LPS-induced IL-10 expression in RAW264.7 cells independent of AhR genomic pathway

**Figure 3:**
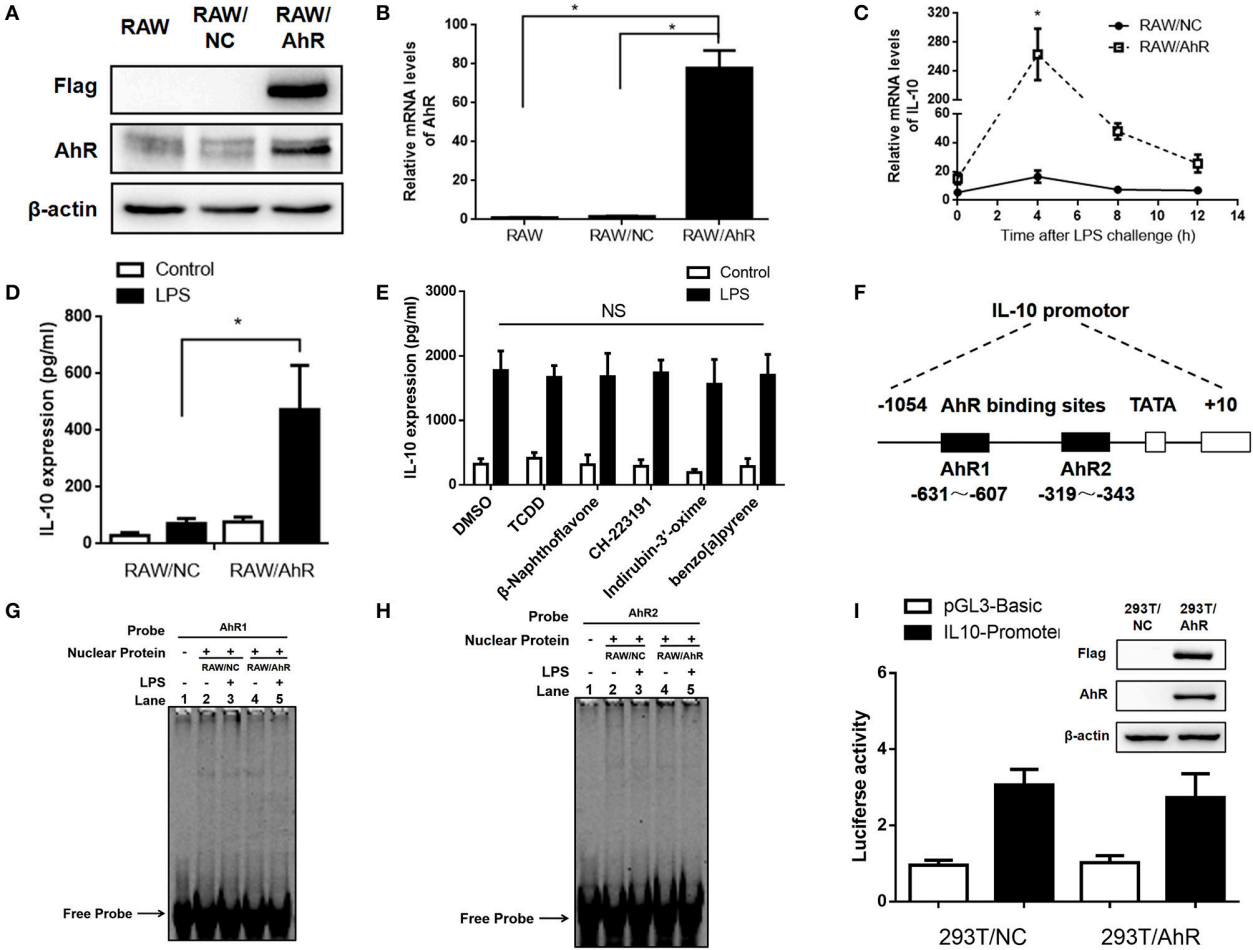
AhR upregulated LPS-induced IL-10 expression in RAW264.7 cells independent of AhR genomic pathway. AhR and Flag protein **(A)** and mRNA **(B)** levels were detected in RAW, RAW/NC, and RAW/AhR cells. The experiment was repeated three times; combined results are compared using one-way ANOVA test (RAW/NC, *P* < 0.001; RAW/AhR, *P* < 0.001). **(C,D)** RAW/NC and RAW/AhR cells were stimulated with LPS at the indicated time points. **(C)** IL-10 mRNA levels were detected with qRT-PCR. The experiment was repeated three times; combined results are compared using independent Student' s *t*-test (*P* = 0.025) **(D)** Supernatants were collected 12 h after LPS stimulation and IL-10 production was measured with ELISA. The experiment was repeated six times; combined results are compared using one-way ANOVA test (*P* = 0.002). **(E)** Peritoneal macrophages were pre-treated with TCDD (1 nM), β-naphthoflavone (1 μM), CH-223191 (10 μM), indirubin-3′-oxime (1 μM), and benzo[a]pyrene (10 μM), respectively, for 1 h and then stimulated with or without LPS for 12 h. IL-10 expression was measured with ELISA. **(F)** Schematic of promoter construct of murine *AhR* gene containing 1054 bp upstream of the transcriptional start site cloned into a luciferase reporter vector. Position of the AhR-binding site are presented spanning −631 to −607 bp and −319 to −343 bp. **(G,H)** RAW/NC and RAW/AhR cells were stimulated with or without LPS for 2 h and nuclear-extract proteins were then prepared and incubated with AhR-binding site probe 1 **(G)** and probe 2 **(H)** of the IL-10 promoter. Binding activity was measured using EMSA. **(I)** 293T/NC and 293T/AhR cells were co-transfected with a luciferase reporter gene construct of the murine IL-10 promoter and pRL-TK control plasmids for 48 h. The cells were lysed and luciferase activity was analyzed. AhR and Flag protein levels were detected in 293T/NC and 293T/AhR cells (right). Western blot and EMSA images are representative of three independent experiments. Data in all bar graphs are mean ± SEM of three independent experiments. ^*^P < 0.05; NS, not significant.

To study the regulatory effect of AhR on LPS-stimulated IL-10 expression, we established a stable AhR-overexpressing RAW264.7 murine macrophage cell line. The infection efficacy was confirmed by expression of labeled Flag protein and GFP in the RAW/AhR cells (Figure 3A). Gene and protein expression levels of AhR were significantly upregulated in RAW/AhR cells compared with RAW/normal control (NC) or RAW cells (Figures 3A,B). Consistent with the AhR-KO data, AhR promoted the expression of IL-10 mRNA at different time points, with a peak at 4 h after LPS treatment (Figure 3C). IL-10 protein levels were higher in the LPS-induced RAW/AhR cells compared with the LPS-induced RAW/NC cells (Figure 3D). To determine if the regulation of IL-10 expression was dependent on the AhR genomic pathway, we treated the macrophages with AhR ligands and confirmed their activity by measuring the cytochrome P4501A expression of AhR target gene. The mRNA levels of CYP1A1 were increased by AhR agonists, such as TCDD, β-Naphthoflavone, Indirubin-3′-oxime, benzo[a]pyrene. AhR antagonist had no significant effect on the CYP1A1 expression, such as CH-22319 (Figure S3). LPS-induced IL-10 expression levels were similar in ligands-treated and DMSO-treated cells (Figure 3E).

To examine the effect of AhR on the IL-10 promotor, we constructed a pGL3-Basic-IL10-promoter-luc plasmid spanning −1054 to +10 bp of the IL-10 promotor. Based on the JASPAR database, the AhR binding site was predicted to be the sequence spanning −631 to −607 bp and −319 to −343 bp (Figure 3F). Binding of nuclear protein to the AhR-binding site of the IL-10 promotor was detected by electrophoretic mobility shift assay (EMSA). Binding activity levels were similar in RAW/AhR and RAW/NC cells (Figures 3G,H). We also established a stable AhR-overexpressing HEK 293T cell line and examined the transcriptional activity of the IL-10 promoter using a luciferase reporter assay. Interestingly, transcriptional activity was also similar in AhR-overexpressing and NC cells (Figure 3I). This indicated that the DNA-binding activity and the transcriptional activity of the IL-10 promoter was unaffected by AhR-overexpression. This analysis thus revealed that AhR upregulated LPS-induced IL-10 expression independent of the AhR genomic pathway.

### Maximal IL-10 production was associated with STAT3 activation in LPS-induced AhR-overexpressing macrophages

**Figure 4:**
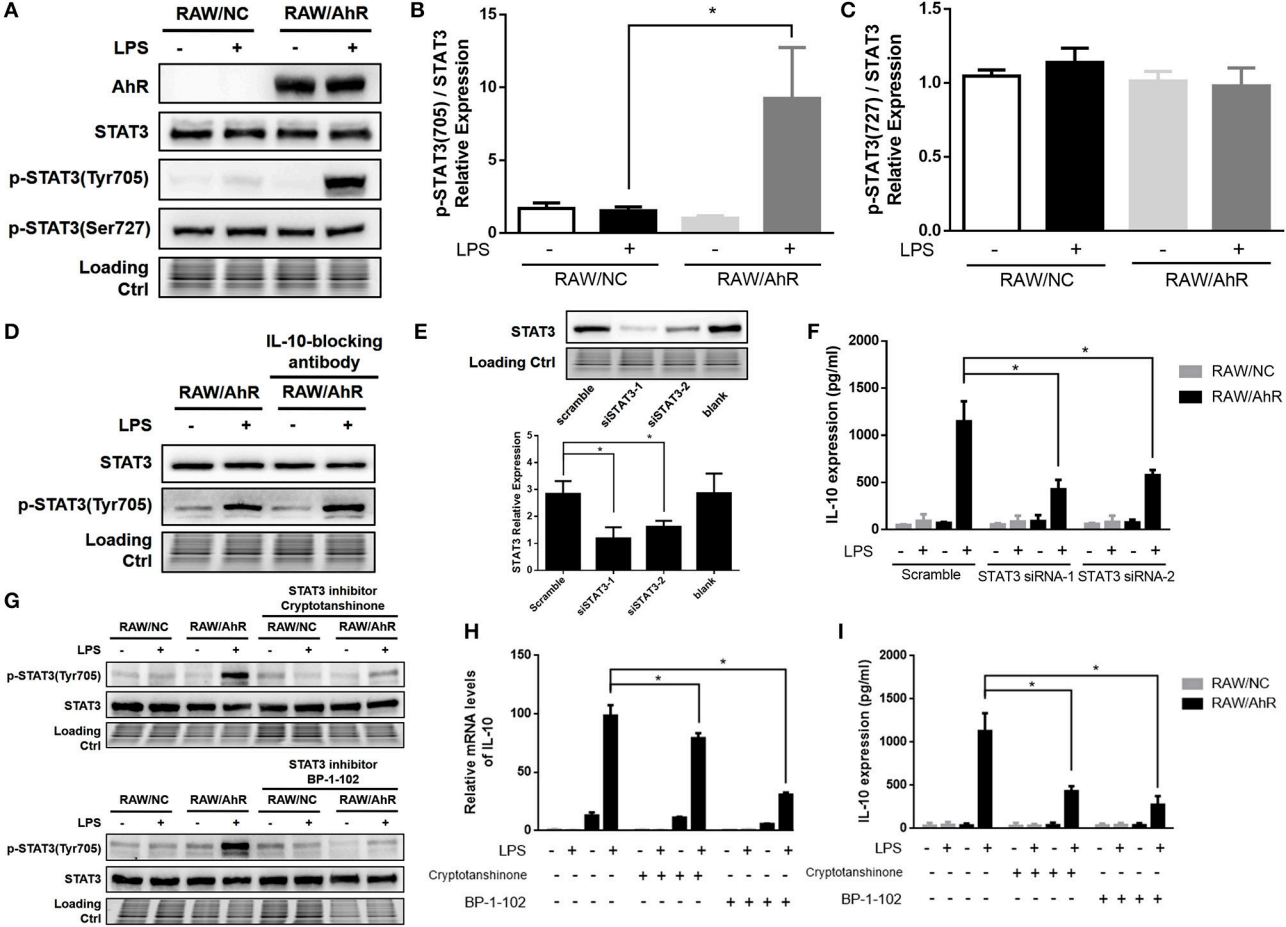
Maximal IL-10 production was associated with STAT3-activation in LPS-induced AhR-overexpressing macrophages. RAW/NC and RAW/AhR cells were untreated or treated with LPS. **(A–C)** Cells were stimulated with LPS for 1 h and cell lysates were used for western blotting analysis with anti-phosphotyrosine STAT3, phosphoserine STAT3, STAT3, and AhR antibodies. **(B,C)** Densitometric analysis of relative abundance of phosphotyrosine-STAT3 and phosphoserine-STAT3 normalized to total STAT3. The experiment was repeated three times; combined results are compared using one-way ANOVA test (*P* = 0.020). **(D)** Cells were pre-treated with IL-10-blocking antibody (1 μg/ml) for 12 h and then stimulated with LPS for 1 h, followed by western blotting of whole-cell extracts. **(E,F)** Cells were transfected with scrambled or STAT3-specific siRNA and then incubated with or without LPS for 12 h. Whole-cell lysates were subjected to western blotting analysis. Combined results are compared using one-way ANOVA test (*n* = 3; siSTAT3-1, *P* = 0.015; siSTAT3-2, *P* = 0.039). IL-10 levels in the supernatant were determined with ELISA. Combined results are compared using one-way ANOVA test (*n* = 3; siSTAT3-1, *P* = 0.004; siSTAT3-2, *P* = 0.009). **(G–I)** Cells were pre-treated with two different STAT3 inhibitors (cryptotanshinone and BP-1-102) for 1 h and then stimulated with or without LPS for the indicated times. **(G)** Whole-cell extracts were immunoblotted with anti-phosphotyrosine STAT3 and STAT3 antibodies. **(H)** IL-10 mRNA levels were detected with qRT-PCR. Combined results are compared using one-way ANOVA test (*n* = 3; cryptotanshinone, *P* = 0.017; BP-1-102, *P* = 0.010). **(I)** IL-10 protein levels were detected with ELISA. Combined results are compared using one-way ANOVA test (*n* = 3; cryptotanshinone, *P* = 0.004; BP-1-102, *P* = 0.002). Western blot images are representative of three independent experiments. Data in all bar graphs are mean ± SEM of three independent experiments. Differences were analyzed with one-way ANOVA. ^*^P < 0.05.

**Figure 5:**
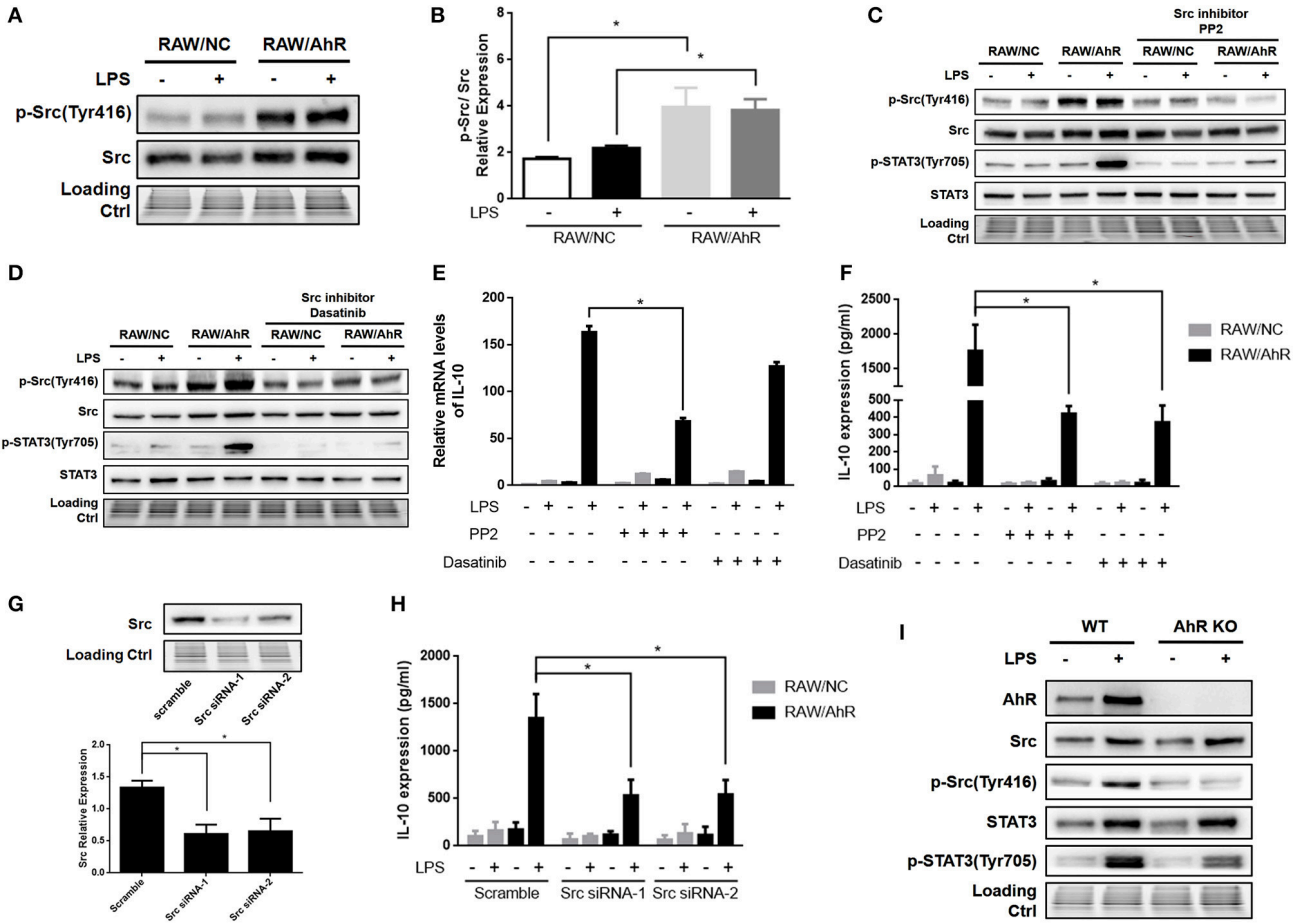
AhR upregulated LPS-induced IL-10 expression through Src pathway in macrophages. **(A,B)** RAW/NC and RAW/AhR cells were untreated or treated with LPS for 1 h and cell lysates then subjected to western blotting analysis with anti-phosphotyrosine Src and Src antibodies. **(B)** Densitometric analysis of relative abundances of phospho-Src normalized to total Src. Combined results are compared using one-way ANOVA test (*n* = 3; control, *P* = 0.012; LPS, *P* = 0.039). **(C–F)** Cells were pre-treated with two different Src inhibitors (PP2 and dasatinib) for 1 h and then stimulated with or without LPS for the indicated times. **(C,D)** Whole-cell extracts were immunoblotted with specific antibodies. **(E)** IL-10 mRNA levels were detected with qRT-PCR. Combined results are compared using one-way ANOVA test (*n* = 3; PP2, *P* = 0.015). **(F)** IL-10 protein levels were detected with ELISA. Combined results are compared using one-way ANOVA test (*n* = 3; PP2, *P* = 0.003; dasatinib, *P* = 0.002). **(G,H)** Cells were transfected with scrambled or Src-specific siRNA and then incubated with or without LPS for 12 h. Whole-cell lysates were subjected to western blotting analysis. Combined results are compared using one-way ANOVA test (*n* = 4; siSrc-1, *P* = 0.003; siSrc-2, *P* = 0.004). IL-10 levels in the supernatant were determined with ELISA. Combined results are compared using one-way ANOVA test (n = 3; siSrc-1, *P* = 0.011; siSrc-2, *P* = 0.011). **(I)** Peritoneal macrophages isolated from WT or AhR-KO mice were stimulated with or without LPS for 6 h. Whole-cell lysates were subjected to western blot analysis. Western blot images are representative of three independent experiments. Data in all bar graphs are mean ± SEM of three independent experiments. Differences were analyzed with one-way ANOVA. ^*^P < 0.05.

STAT3 is a key regulator of LPS-stimulated IL-10 prodution. We therefore determined if STAT3 level and STAT3 phosphorylation were involved in the regulation of IL-10 expression by AhR. Tyrosine phosphorylation of STAT3 was enhanced in RAW/AhR compared with in RAW/NC cells after LPS stimulation, but STAT3 serine phosphorylation of STAT3 was unaffected by AhR (Figures 4A–C). Likewise, LPS-stimulated STAT3 phosphorylation was decreased in peritoneal macrophages from AhR-KO mice compared with WT mice (Figure 5I). To determine if STAT3 phosphorylation was dependent on IL-10 autocrine secretion by RAW/AhR cells, we pretreated the cells with an IL-10-blocking antibody in the culture medium before LPS stimulation. IL-10 expression was almost undetectable in LPS-induced RAW/AhR cells incubated with an IL-10-blocking antibody, but tyrosine phosphorylation of STAT3 was not dependent on the presence of IL-10 in the culture medium (Figure 4D). STAT3 activation appeared to be regulated by the Akt/mTOR/p70S6 and MAPK pathways, but Akt, mTOR and p70S6 levels were unaffected by AhR (Figure S4B). There were no significant differences in the main MAPK pathway factors between RAW/NC and RAW/AhR cells (Figure S4A). Furthermore, IL-10 expression was unaffected by a MEK inhibitor (Figures S4C,D). We therefore established that STAT3 activation was independent of the Akt/mTOR/p70S6 and MAPK pathways (Figure S4).

To probe the role of STAT3 in this context, we silenced STAT3 using two different siRNAs (STAT3 siRNA-1 and STAT3 siRNA-2) in RAW/NC and RAW/AhR cells (Figure 4E). LPS-induced IL-10 expression was decreased in RAW/AhR cells treated with either STAT3 siRNA compared with cells treated with scrambled siRNA (Figure 4F). We also determined if inhibition of STAT3 affected IL-10 production using the STAT3 inhibitors (cryptotanshinone and BP-1-102), which significantly inhibited tyrosine phosphorylation of STAT3 (Figure 4G). LPS-stimulated IL-10 mRNA (Figure 4H) and protein expression levels (Figure 4I) were reduced in RAW/AhR cells treated with STAT3 inhibitor compared with control cells. These results supported our hypothesis that AhR upregulated LPS-induced IL-10 expression via the STAT3 activation.

### AhR upregulated LPS-induced IL-10 expression through Src pathway in macrophages

Src kinase is known to mediate the tyrosine-phosphorylation of intracellular target proteins (19), and its activity is frequently accompanied by AhR activation (9, 34). Notably, Src tyrosine phosphorylation was enhanced in RAW/AhR compared with RAW/NC cells (Figures 5A,B). Src activity required AhR, which was necessary for IL-10 expression. Src kinase activity was downregulated by the selective Src inhibitors (PP2 and dasatinib), which significantly inhibited Src tyrosine phosphorylation (Figures 5C,D). LPS-stimulated IL-10 mRNA (Figure 5E) and protein (Figure 5F) were reduced in RAW/AhR cells treated with Src inhibitor compared with control cells. To demonstrate the role of Src in IL-10 expression, we silenced Src in RAW/NC and RAW/AhR cells using two different Src siRNAs (Src siRNA-1 and Src siRNA-2) (Figure 5G). LPS-induced IL-10 expression was decreased in RAW/AhR cells treated with either siRNAs compared with cells treated with scrambled siRNA (Figure 5H). Notably, STAT3 tyrosine phosphorylation was also inhibited by Src inhibitors, which showed that STAT3 activity was triggered by Src activation (Figures 5C,D). Likewise, LPS-stimulated Src phosphorylation was decreased in peritoneal macrophages from AhR-KO mice compared with WT mice (Figure 5I). Moreover, we found no direct interaction between AhR and Src-STAT3 in inflammatory macrophages (Figure S5). These results indicated that AhR-associated Src activity was responsible for STAT3 phosphorylation and IL-10 production by inflammatory macrophages.

### Adoptive transfer of AhR-expressing peritoneal macrophages protected mice against LPS-induced peritonitis

**Figure 6:**
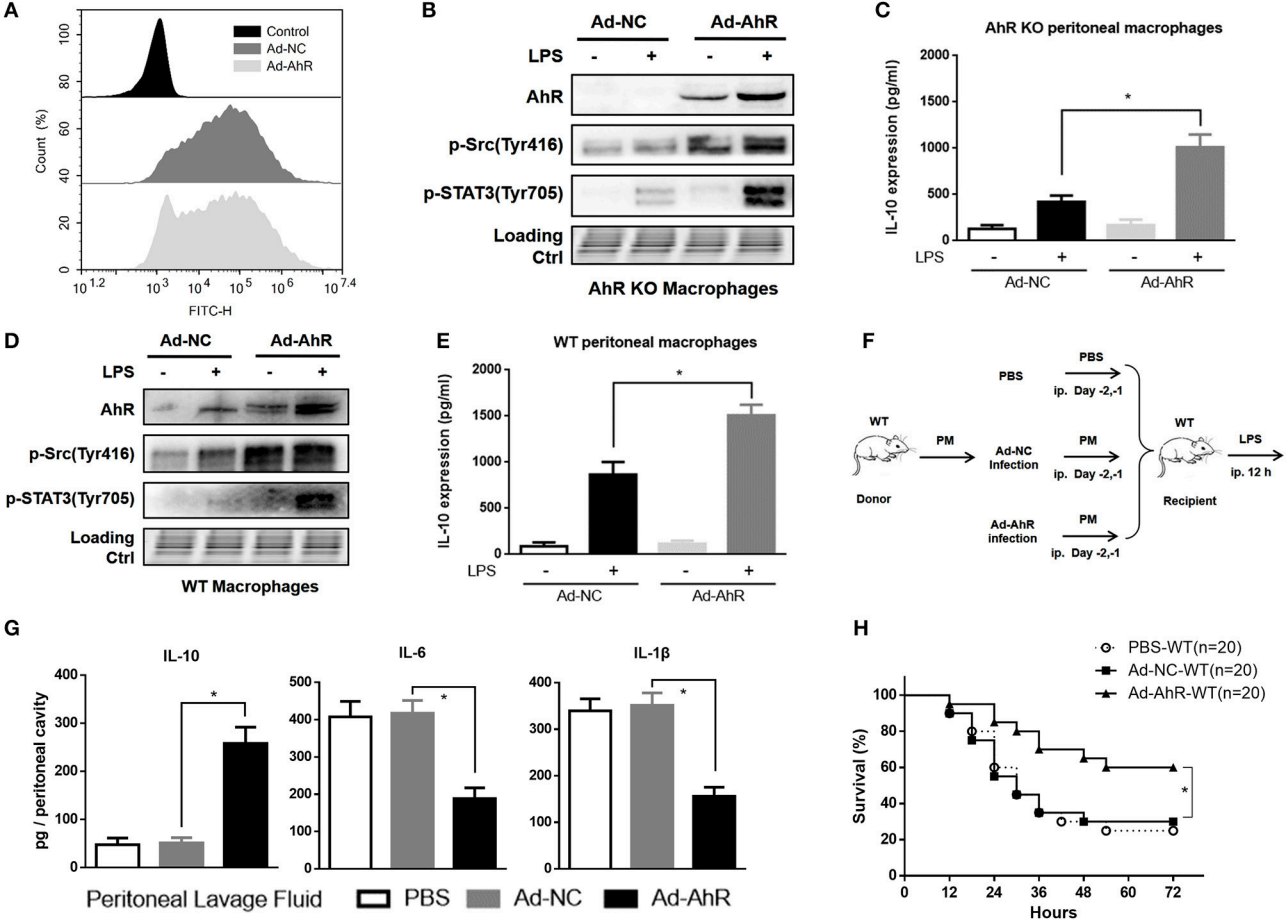
Adoptive transfer of AhR-expressing peritoneal macrophages protected mice against LPS-induced peritonitis. **(A)** Flow cytometry profiles of peritoneal macrophages after 36 h incubation with AhR-overexpressing adenovirus (Ad-AhR) or negative control adenovirus (Ad-NC). **(B–E)** Peritoneal macrophages from AhR-KO **(B,C)** and WT **(D,E)** mice were transfected with Ad-AhR or Ad-NC and then incubated with or without LPS for 12 h. Whole-cell lysates were subjected to western blotting analysis and IL-10 levels in the supernatant were determined with ELISA. Combined results are compared using one-way ANOVA test (*n* = 3; AhR-KO, *P* < 0.001; WT, *P* = 0.001). **(F)** Peritoneal macrophages transfected with Ad-NC or Ad-AhR were injected intraperitoneally into WT mice at 2 days before the start of LPS treatment. **(G)** Peritoneal lavage fluid was collected from each group at 12 h after LPS stimulation and IL-10 (*n* = 6; *P* < 0.001), IL-6 (*n* = 6; *P* < 0.001), and IL-1β (*n* = 6; *P* < 0.001) protein levels were measured with ELISA.**(H)** Survival rates were monitored for 72 h after LPS stimulation and presented as Kaplan–Meier survival curves. The combined results were analyzed with the log-rank test (*n* = 20; *P* = 0.003). Data in all bar graphs are mean ± SEM of three independent experiments. Differences were analyzed with one-way ANOVA. ^*^P < 0.05.

To enhance the ability of IL-10 production in peritoneal macrophages, the recombinant AhR-expressing adenovirus (Ad-AhR), and negative control adenovirus (Ad-NC) were constructed and transfected into AhR-KO and WT macrophages. The mean fluorescence intensity of FITC was increased in adenovirus-treated cells compared with control cells (Figure 6A). Compared with Ad-NC, Ad-AhR significantly improved the phosphorylation of Src and STAT3 in AhR-KO peritoneal macrophages (Figure 6B), as well as increased amounts of IL-10 protein (Figure 6C). We also found that the levels of AhR, p-Src, and p-STAT3 were enhanced after WT peritoneal macrophages were infected with Ad-AhR compared with Ad-NC (Figure 6D). IL-10 protein levels were also increased from Ad-AhR treated group compared with Ad-NC treated group after LPS stimulation (Figure 6E). To determine if AhR-expressing PMs could suppress the development of LPS-induced peritonitis, the PMs transfected with Ad-AhR or Ad-NC were adoptively transferred into WT mice after intra-peritoneal injection of LPS (Figure 6F). Adoptive transfer of Ad-AhR transfected PMs but not Ad-NC or PBS significantly increased the IL-10 expression and suppressed IL-6 and IL-1β expression in peritoneal lavage fluid from WT peritonitis mice (Figure 6G). Furthermore, compared with PBS or Ad-NC, Ad-AhR significantly improved the survival of mice in the lethal LPS-induced peritonitis model (Figure 6H). These results demonstrated that the adoptive transfer of AhR-expressing PMs protected mice against LPS-induced peritonitis through high IL-10-production.

## Discussion

The results revealed that LPS-induced AhR expression was mediated by the NF-κB pathway in macrophages. Previous data reported that AhR was increased in naïve T cells stimulated by TGF-β plus IL-6, but not by either of these factors alone (31). AhR was also expressed in BMDCs stimulated by both LPS and CpG (32). LPS-induced both AhR mRNA and protein expression were increased in human monocyte-derived DC (33). Besides these cells, we also confirmed that LPS-induced AhR expression was increased in peritoneal macrophages, BMDMs and spleen macrophages. This provides the first evidence for systemic AhR expression in inflammatory macrophages. Furthermore, we demonstrated that LPS-induced AhR expression was disturbed by preincubation of the cells with PDTC, consistent with previous findings showing that the human AhR gene was regulated via the NF-κB signaling pathway (33). In addition, numerous studies have indicated the physiological role of AhR in limiting the inflammatory response (2, 3). These results provide new insights into that AhR is highly expressed after LPS stimulation in innate immune response, which represents feedback control of the inflammatory response through negative regulation of TLRs signaling pathway by AhR (4, 35).

There is considerable evidence to suggest that AhR signaling is involved in immune system function and recent studies have described a complex interplay between AhR signaling and inflammatory mediators (36, 37). AhR could downregulate the expression of IL-6 (13, 14) and IL-1β (15) in inflammatory macrophages, in line with our findings. In this study, LPS-induced macrophages isolated from AhR-KO mice shown impaired expression of IL-10, while IL-10 expression was significantly elevated in LPS-induced AhR-overexpressing RAW264.7 cells compared with NC cells. Consistent with our results obtained in mouse inflammatory macrophages, AhR has previously been shown to regulate IL-10 expression in different types of immune cells, including human Treg cells (38), regulatory type 1 cells (39), mast cells (40), and natural killer cells (41). In the presence of LPS or CpG, AhR-KO mature BMDCs induced immune responses characterized by reduced Kyn and IL-10 production (32). Additionally, the potent AhR ligand TCDD increased IL-10 expression in LPS-stimulated BMDCs (42, 43), and this effect was suppressed by the AhR antagonist α-naphthoflavone (42). These findings suggested that AhR was required to induce IL-10 expression in the innate and adaptive immune responses.

To further examine whether AhR modifies macrophage polarization, we evaluate M1/M2 markers in LPS-induced macrophages. AhR-overexpressing cells exhibited a weaker M1 polarization as revealed by cytokines production, and lower levels of IL-1β, IL-6, NOS2, IL-12p40, IL-23 were observed after LPS-mediated activation. As to M2 markers, we only found IL-10 mRNA level was higher in AhR-overexpressing cells. No differences were observed in TNF-α, TGF-β, and Arg1 levels between RAW/NC and RAW/AhR cells. The above data indicate that AhR-overexpressing cells present a mixed response after LPS stimulation. Previous evidence also showed that disruption of the AhR gene alters macrophage polarization to either the M1 or M2 phenotype compared to WT macrophage (44). These findings showed the important role of AhR in macrophage polarization.

AhR is a ligand-dependent protein activated by a structurally diverse array of ligands. The AhR canonical pathway that is also called as AhR genomic pathway is characterized by AhR nuclear translocation. However, we identified that the AhR chaperone proteins ARNT and HSP90 were not involved in the regulation of inflammatory macrophages, and LPS-induced IL-10 expression was unaffected by most AhR ligands, which bound to AhR to regulate the downstream genomic signaling pathway. Several studies reported that TCDD-driven AhR activation resulted in significantly increased IL-10 expression (38, 39, 42, 43), and that AhR and ARNT were required for optimal production of IL-10 (41). However, in the absence of a ligand, we also found that elevated AhR could upregulate LPS-induced IL-10 expression independent of the AhR genomic pathway. The DNA-binding activity at the AhR-binding site of the IL-10 promoter was similar in nuclear extracts from RAW/AhR compared with RAW/NC cells. Interestingly, transcriptional activity was also similar in AhR-overexpressing and NC cells according to a luciferase reporter assay. We demonstrated that AhR did not directly affect the transcriptional activity of L-10 promoter. This suggested that other AhR-associated transcription factors might be implicated in the regulation of IL-10 gene expression in inflammatory macrophages.

Previous evidences showed that STAT3 activation regulated downstream genes by AhR ligands including TCDD (45), indoxyl sulfate (46), and β-naphthoflavone (47). However, the current results showed that ligand-independent activation of AhR could efficiently mobilize LPS-stimulated STAT3. STAT3 protein was phosphorylated at tyrosine, but not at serine, and LPS-stimulated STAT3 phosphorylation was decreased in AhR-KO macrophages. AhR-regulated IL-10 production was attenuated by specific STAT3 inhibitors and siRNAs, suggesting that LPS-induced IL-10 expression was associated with the AhR-STAT3 pathway in macrophages. This was consistent with the finding that AhR cooperated with STAT3 to regulate the differentiation of both Th17 and Treg cells (48) and the IL-22 promoter in CD4+ T cells (49). However, immunoprecipitation revealed no direct interaction between AhR and STAT3. Notably, STAT3 has been implicated in the feed-forward loop for IL-10 expression. Our results showed that tyrosine phosphorylation of STAT3 was unaffected by an IL-10-blocking antibody, suggesting that AhR-associated STAT3 activation was independent of autocrine IL-10 signaling. Furthermore, we established that STAT3 activation was independent of the Akt/mTOR/p70S6 and MAPK pathways.

AhR-dependent activation of Src kinase was confirmed in two cell lines (34), and the importance of Src kinase after AhR-ligands treatment has been in terms of various other effects (50, 51). The present findings suggested that AhR-overexpression was required for tyrosine phosphorylation of Src, and its activity was enhanced in an AhR ligand-independent manner. AhR-KO fibroblasts showed altered FAK phosphorylation and impaired Src activation (52), in line with our findings indicating that Src kinase activity was influenced by changes in AhR expression levels. In unstimulated cells, Src and AhR coexist in a protein complex that may also contain HSP90, AhR-interacting protein, and P23 (53). However, we found no direct interaction between AhR and Src in inflammatory macrophages. AhR-regulated IL-10 production was attenuated by specific Src inhibitors and siRNAs, and STAT3 phosphorylation was also inhibited by Src inhibitors, indicating that Src acted upstream of STAT3 in the induction of IL-10. In support of this hypothesis, AF1q-induced platelet-derived growth factor receptor signaling enhanced STAT3 activity through Src kinase activation, which could be blocked by the Src kinase inhibitor PP1 (54). Moreover, inhibition of Src by PP2 antagonized the activation of STAT3 conferred by GRP78-induced epidermal growth factor signaling (55). These and our current results suggested that AhR-associated Src activity was responsible for STAT3 phosphorylation and IL-10 production by inflammatory macrophages.

Our results showed that the adoptive transfer of high-IL-10-producing macrophages significantly suppressed the inflammatory response, and improved survival in mice with LPS-induced peritonitis. AhR was previously shown to be involved in the regulation of inflammatory diseases, and accumulating evidence has suggested the existence of multiple crosstalk between AhR and inflammation. AhR-KO mice were hypersensitive to LPS-induced septic shock and showed markedly increased plasma levels of IL-1β, IL-18, IL-6, and TNF-α (8), and activated AhR was shown to play a central role in limiting endotoxin-triggered inflammation (9). We speculated that the pathological process might involve a regulatory effect of AhR on IL-10. Intriguingly, we found that adoptive transfer of AhR-expressing PMs protected mice against LPS-induced peritonitis via increased IL-10-production. These results strongly indicate that AhR enhances IL-10 production by macrophages, resulting in suppression of the inflammatory response. Further studies will be needed to investigate the effect of AhR on IL-10 in other inflammatory diseases.

This study had some limitations. Although we established that LPS-induced AhR expression was increased by the NF-κB pathway in macrophages, it remains unclear if AhR is also highly expressed after stimulation by other TLR-ligands in the innate immune response. Furthermore, we showed that AhR regulated IL-10 expression through a non-genomic pathway associated with Src- and STAT3-activation, but the details of this interaction remain to be fully characterized. Further studies are needed to track the location of AhR and detect the AhR-interacting proteins. It is also possible that mechanisms other than the Src-STAT3 signaling pathway may be involved in regulating IL-10 expression in other situations, and more studies are required to clarify this. Nevertheless, the present results provide valuable information about the crosstalk between AhR and IL-10 expression in macrophages and establish an innovative platform for future studies of the mechanisms of AhR in immune regulation.

**Figure 7:**
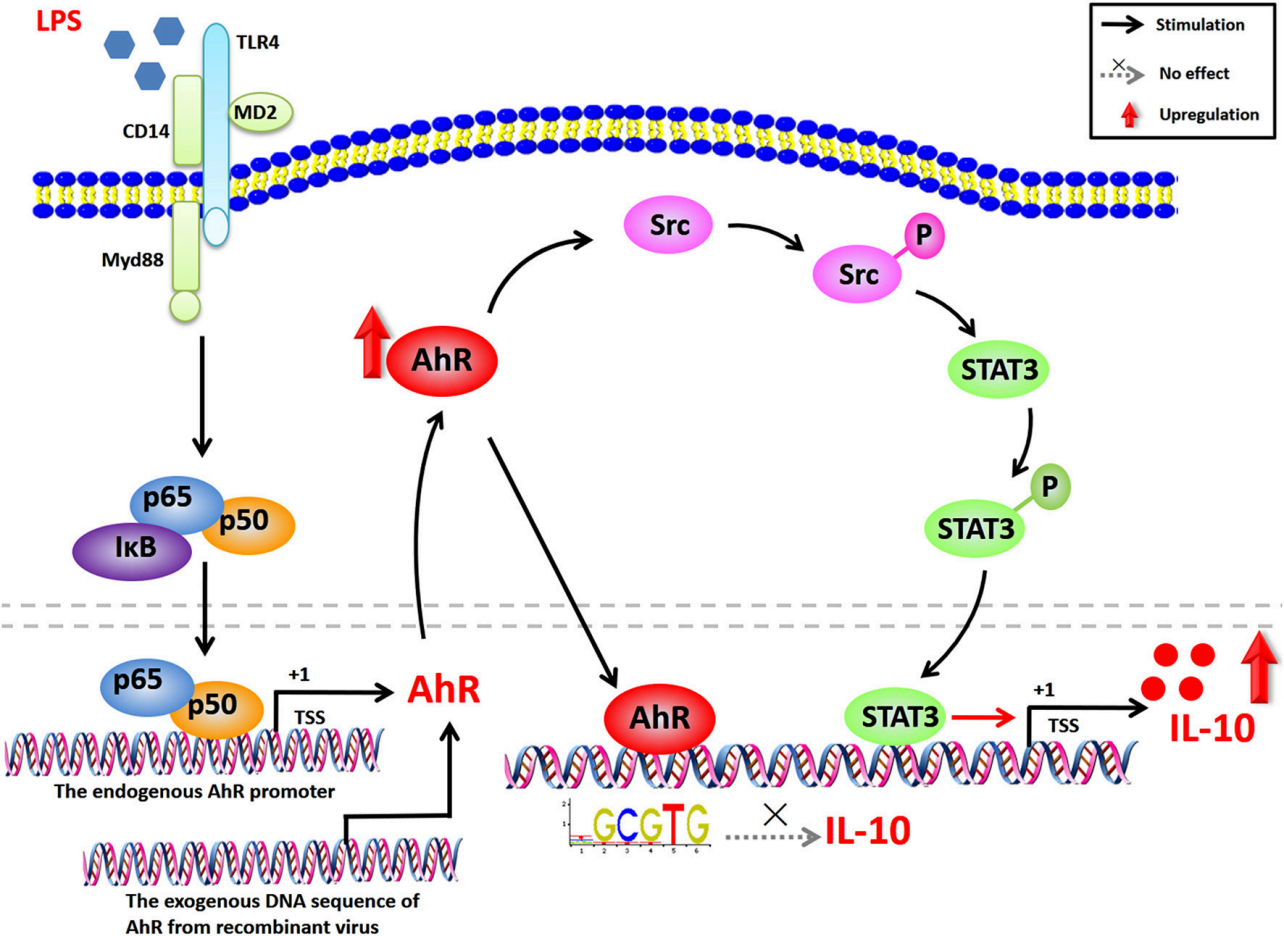
Proposed network for AhR-Src-STAT3-IL-10 signaling pathway in inflammatory macrophages. AhR expression is highly enhanced in LPS-induced inflammatory macrophages via the NF-κB pathway. AhR binds to the IL-10-promoter, but does not affect the transcriptional activity of IL-10. Increased AhR in the cytoplasm upregulates the tyrosine phosphorylation of Src, which can be suppressed by selective Src inhibitors (PP2 and dasatinib). STAT3 is also activated by phosphorylation of tyrosine 705, triggered by Src tyrosine kinase and suppressed by selective STAT3 inhibitors (cryptotanshinone and BP-1-102). STAT3 activation is independent of IL-10 autocrine feedback, Akt/mTOR, and MAPK pathways. The activated signaling pathway promotes LPS-induced IL-10 expression. Overall, AhR-associated Src activity is responsible for the tyrosine phosphorylation of STAT3 and IL-10 expression in LPS-induced inflammatory macrophages.

In the current study, we showed that AhR was markedly increased in LPS-stimulated macrophages, and found that AhR positively regulated the expression of IL-10 in macrophages and RAW264.7 cells. Maximal LPS-stimulated IL-10 production was associated with Src and STAT3 activation, but not via the ligand activation and the AhR genomic pathway. Furthermore, AhR-associated Src activity was responsible for tyrosine phosphorylation of STAT3 and IL-10 expression in inflammatory macrophages (Figure 7). In conclusion, our results indicate that the AhR-Src-STAT3-IL-10 signaling pathway is a critical pathway involved in the immune regulation of inflammatory macrophages and suggest that AhR may be a potential therapeutic target in immune response. Further understanding and rational utilization of this signaling pathway may help in the establishment of effective therapies for inflammatory diseases.

## Materials and methods

### Materials

LPS (Escherichia coli 0111: B4), TCDD, β-naphthoflavone, CH-223191, indirubin-3′-oxime, and benzo[a]pyrene were purchased from Sigma-Aldrich. PE anti-mouse F4/80 Antibody (123110), FITC anti-mouse/human CD11b Antibody (101206) and LEAF™-Purified anti-mouse IL-10-blocking Antibody (JES5-16E3) were obtained from Biolegend. The NF-κB inhibitor PDTC, STAT3 inhibitors cryptotanshinone and BP-1-102, the Src inhibitors PP2 and dasatinib, and MEK inhibitor PD98059 were purchased from Selleck. Penicillin, streptomycin, RPMI 1640 and fetal bovine serum were produced by Gibco-BRL Invitrogen.

### Macrophage preparation and cell culture

Peritoneal macrophages were obtained from mice by flushing the peritoneal cavity with 5 ml PBS 4 days after intraperitoneal injection of 4% thioglycollate. The peritoneal exudate cells were centrifuged (300 × g) and adhered to the Petri dish for 2 h at 37°C. Non-adherent cells were removed by gently washing three times with warm PBS, and peritoneal macrophages were then harvested from the adherent cells. Murine bone marrow cells were obtained from mice and differentiated into mature macrophages during 7 days culture in murine complete medium supplemented with GM-CSF (25 ng/ml; Miltenyi Biotec). BMDMs were harvested from the adherent cells. Isolated spleens were ground in saline solution using a glass homogenizer and the cell suspension was passed through cell strainer (70 μm; Corning). The resulting suspension of splenocytes was centrifuged (300 × g, 5 min) and resuspended in red cell lysis buffer (Tiangen) for 5 min. The cell suspension was then incubated in the Petri dish for 2 h at 37°C. The cell monolayer was flushed with PBS to remove attendant lymphocytes, fibroblasts, and erythrocytes. Spleen macrophages were obtained using a scraper and used for further experiments.

RAW264.7 cells (ATCC®-TIB-71™) and HEK 293T cells (ATCC®-CRL-11268™) were maintained in RPMI1640 and DMEM medium, respectively, supplemented with 100 U/ml penicillin, 100 mg/ml streptomycin, and 10% FBS. The cells were grown at 37°C and 5% CO_2_ in humidified air. RAW264.7 cells and 293T cells were stably transfected with recombinant lentivirus containing the whole coding sequence of AhR-EGFP (GeneChem). The stable AhR-overexpressing RAW264.7 cell line (RAW/AhR) and normal control cell line (RAW/NC) were maintained in the presence of 2 μg/ml puromycin.

### Mice

All animal experiments were carried out in accordance with the recommendations of the guidelines for Care and Use of Laboratory Animals of the National Institutes of Health, and the following animal protocol was approved by the intramural Committee on Ethics Conduct of Animal Studies of the Army Medical University. AhR+/− mice lacking exon 2 on a C57BL/6 background were purchased from the Jackson Laboratory. AhR+/+ and AhR−/− mice were bred from AhR+/− mice and maintained as separate colonies. C57BL/6 mice (8–10 weeks old, 18–22 g) were purchased from the Experimental Animal Center of Army Medical University (Chongqing, China).

### Flow cytometry

Peritoneal macrophages, BMDMs and spleen macrophages were isolated from mice and were prepared for single-cell suspensions. The cells were stained with conjugated fluorescent antibodies at predetermined optimum concentrations (anti-F4/80-PE and anti-CD11b-FITC) and incubate on ice for 20 min in the dark. After washing and centrifugation, the cells were resuspended in PBS and subjected to flow cytometric analysis.

### Elisa

Supernatants were collected from treated cells and peritoneal lavage fluid and stored at −80°C until analysis. Expression levels of IL-10, IL-6, and IL-1β were assessed using ELISA kits (Boster). The absorbance was measured at 450 nm.

### Quantitative reverse transcription PCR analysis

Total RNA was extracted using TRIzol reagent (Invitrogen). Complementary DNA was synthesized from 1 μg of total RNA by reverse transcription, and the mRNAs of interest were quantitated by qRT-PCR analysis using SYBR Premix (TaKaRa) on a BioRad CFX96. β-actin was chosen as the reference gene (Table 1).

**Table 1:** Primers used to amplify mRNAs via qRT-PCR.

**Gene**	**Forward primer (5^′^-3^′^)**	**Reverse primer (5^′^-3^′^)**
AhR	CAAATCCTTCTAAGCGACACAG	TGACGCTGAGCCTAAGAACA
IL-10	GCTCTTACTGACTGGCATGAG	CGCAGCTCTAGGAGCATGTG
IL-6	ACCACGGCCTTCCCTACTTC	CTCATTTCCACGATTTCCCAG
IL-1β	GCAACTGTTCCTGAACTCAACT	ATCTTTTGGGGTCCGTCAACT
TNF-α	GAGTCCGGGCAGGTCTACTTT	CAGGTCACTGTCCCAGCATCT
TGF-β	CACAGAGAAGAACTGCTGTG	AGGAGCGCACAATCATGTTG
CYP1A1	GCAATGAGTTTGGGGAGGT	AAGGCATCCAGGGAAGAGTT
IL-12p40	TGGTTTGCCATCGTTTTGCTG	ACAGGTGAGGTTCACTGTTTCT
IL-23	ATGCTGGATTGCAGAGCAGTA	ACGGGGCACATTATTTTTAGTCT
NOS2	GTTCTCAGCCCAACAATACAAGA	GTGGACGGGTCGATGTCAC
Arg1	GAGATTCCTGAAACCGAACCTC	ACTACAGGGATTACTGTCGAGG
β-actin	AGCCATGTACGTAGCCATCC	CTCTCAGCTGTGGTGGTGAA

### Luciferase reporter assay

HEK 293T cells were co-transfected with 1 μg of pGL3-Basic-IL10-promoter-luc and 0.1 μg of pRL-TK plasmid (Fitgene Biotech) for 48 h using Lipofectamine 2000 transfection reagent (Invitrogen). The cells were then lysed and detected using dual luciferase reporter gene assay kit (Beyotime). IL-10 transcription activity was defined as the percentage of relative light units of firefly luciferase to *Renilla* luciferase.

### EMSA

Nuclear extracts from treated RAW 264.7 cells were prepared using a nuclear and cytoplasmic protein extraction kit (Beyotime) and quantified. Binding activity between nuclear proteins and the AhR-binding site of the *IL-10* promoter was assessed by electrophoresis of the DNA–protein complexes on a 6% polyacrylamide native gel for 40 min at 180 V, followed by detection at 700 nm using the Odyssey scanning bed (LiCor). The probes labeled infrared ray 700 (Viagene Biotech), containing the consensus recognition site for AhR, were as follows: AhR1 forward: 5′- GCTTCTTCGTGAAACACGGGGCAGA-3′; AhR1 reverse: 5′- TCTGCCCCGTGTTTCACGAAGAAGC-3′; AhR2 forward: 5′- GACCTGGGAGTGCGTGAATGGAATC-3′; AhR2 reverse: 5′-GATTCCATTCACGCACTCCCAGGTC-3′.

### Western blotting

Cells were lysed in lysis buffer (50 mM Tris-HCl [pH 7.5], 150 mM NaCl, 1% Triton X-100, 5 mM EDTA, 0.5 mM Na_3_VO_4_, 50 mM NaF, 1 mM PMSF, and protease inhibitor cocktail [Beyotime]) for 30 min on ice. Cell debris was removed by centrifugation at 12,000 × g for 15 min at 4°C. Protein concentrations in the cell lysates were determined using a BCA protein assay kit (Beyotime). An equal amount of protein for each sample was separated on 10% Tris-Glycine extended stain-free polyacrylamide gels (Bio-Rad) and the loading control was measured by imaging of the stain-free gel. Total protein stains were used as loading control for quantitative protein analysis. SDS gels were then transferred to polyvinylidene difluoride membranes (Millipore), blocked with 5% skim milk for 1 h in Tris buffered saline contained 0.1% Tween 20, and incubated with the indicated primary antibodies overnight at 4°C. The next day, the membranes were washed and probed for 1 h with the respective HRP-conjugated secondary antibody (Cell Signaling Technology). The immunoreactive bands were detected using an enhanced chemiluminescence detection system (Bio-Rad), and the band intensities were measured by densitometric analysis using Image J software. Anti-AhR (BML-SA210) antibody was purchased from Enzo Life, and antibodies against HSP90 (4877), ARNT (5537), FLAG (14793), β-actin (4970), serine-phosphorylated STAT3 (9134), tyrosine-phosphorylated STAT3 (9145), total STAT3 (4904), tyrosine-phosphorylated Src (6943), total Src (2109), p65 (8242), p50 (12540), mTOR (2972), phospho-mTOR (2971), p70S6 (2708), phospho-p70S6 (9204), Akt (9272), total ERK1/2 (4695) and phospho-Erk1/2 pathway sampler kit (9911) were obtained from Cell Signaling Technology.

### Immunofluorescence and confocal analysis

The cells on coverslips (Nest) were fixed with 4% paraformaldehyde (Beyotime) in PBS for 30 min at room temperature, permeabilized with 0.5% Triton X-100 (Beyotime) in PBS for 10 min at room temperature, and then blocked with PBS containing 1% BSA and 1% goat serum for 30 min. The cells on coverslips were incubated with primary antibody to AhR (1:100, sc-8088, Santa Cruz) at 4°C overnight, followed by Alexa Fluor 594-conjugated donkey anti-goat IgG (1:200, Invitrogen) in the dark for 1 h in PBS containing 1% BSA and 0.5% Triton X-100, followed by DAPI (Beyotime) for 10 min. All incubations were performed followed by three washes with PBS. Coverslips were mounted with antifade mounting medium (Beyotime), placed on glass slides, and imaged using a LSM 780 laser-scanning confocal microscope (Carl Zeiss).

### RNA interference

STAT3-specific, Src-specific, and scrambled control siRNAs were obtained from Ribobio. Cells were plated at a density of 2 × 10^5^ cells per well on six-well plates and transfected with 100 nM of STAT3-specific, Src-specific, or scrambled control siRNA in riboFECT™ CP Reagent (Ribobio). Six hours after transfection, siRNA and oligofectamine mixtures were discarded and substituted with RPMI 1640 containing 10% serum, and then incubated with or without LPS for 12 h. Cell lysate and supernatant were collected for analysis 72 h after transfection for analysis. The following siRNA sequences were used: STAT3 siRNA-1: 5′-CCACGTTGGTGTTTCATAA-3′; STAT3 siRNA-2: 5′-GCAGGATCTAGAACAGAAA-3′; Src siRNA-1: 5′-GGCTGCAGATTGTCAATAA-3′; Src siRNA-2: 5′-GCCTCTCTGTATCCGACTT-3′.

### Inhibitor assay

RAW cells (2 × 10^6^ cells/well) were cultured in six-well plates for 12 h and pre-treated with or without cryptotanshinone (10 μM), BP-1-102 (5 μM), PP2 (5 μM), dasatinib (1 μM), and PD98059 (10 μM), respectively, for 1 h. The cells were then stimulated with or without LPS (10 μg/ mL) for the indicated times. Cell lysates were collected for western blotting analysis. IL-10 mRNA and protein levels were detected with qRT-PCR and ELISA, respectively.

### Infection of macrophages with recombinant AhR adenovirus

Recombinant adenovirus containing the gene coding full-length mouse AhR and control adenovirus containing the enhanced GFP gene were obtained from Hanbio Biotechlogy. Recombinant AhR adenovirus infection of peritoneal macrophages were carried out at MOIs of 1000 for 6 h, then cultured for 36 h in fresh medium. The control adenovirus was used as negative control. Twenty-four hours after viral infection, the macrophages were treated with LPS for 12 h and collected for flow cytometry analysis.

### LPS-induced peritonitis model

To induce acute peritonitis, mice were injected intraperitoneally with 10 mg/kg LPS. The transfected PMs (2 × 10^6^) were injected intraperitoneally into WT mice 2 days before LPS-induced peritonitis. The treated mice were sacrificed at 12 h after LPS injection and peritoneal lavage fluid was prepared by a single washing of the peritoneal cavity with 3 ml PBS at 4°C. The IL-10, IL-6, and IL-1β protein concentrations in the peritoneal lavage fluid were quantified with ELISA. In the survival studies, the mice were injected with 20 mg/kg LPS intraperitoneally and were observed for 3 days.

### Statistical analysis

Data were presented as mean ± standard error (SEM). Statistical analyses were performed using one-way ANOVA followed by LSD multiple-comparison test. Survival rates were analyzed using the Mantel–Cox test. All statistical analyses were performed using SPSS 16.0 (SPSS Inc.) or Prism 6.0 (GraphPad Inc.) software, and values of *P* < 0.05 were considered statistically significant.

## Ethics statement

Animal welfare and experimental procedures were conducted in accordance with the guidelines for laboratory animal care of the National Institutes of Health and Army Medical University.

## Author contributions

JZ and LL performed most of the experiments, analyzed the research data, and wrote the manuscript; SY helped with western blotting analysis, luciferase reporter assay, and ELISA; LT helped with lentiviral work and EMSA; LL assisted in siRNA and inhibitor experiments; JiY, XY, and XZ performed animal experiments; WT and XM assisted with qRT-PCR assay; WM helped with western blotting analysis; SC, XF, JuY, and XX contributed to experimental design; HL and CL designed experiments, interpreted data and supervised the work.

### Conflict of interest statement

The authors declare that the research was conducted in the absence of any commercial or financial relationships that could be construed as a potential conflict of interest.

## Abbreviations

AhR: aryl hydrocarbon receptor
ARNT: AhR nuclear translocator
BMDM: bone marrow derived macrophage
DC: dendritic cell
EMSA: electrophoretic mobility shift assay
HSP90: heat shock protein 90
NC: normal control
PDTC: pyrrolidinedithiocarbamate
PM: peritoneal macrophage
TCDD, 2, 3, 7: 8-tetrachlorodibenzo p-dioxin.

## References

[1] Anderson P. Post-transcriptional regulons coordinate the initiation and resolution of inflammation. Nat Rev Immunol. (2010) 10:24–35. 10.1038/nri2685 20029446

[2] Esser C Rannug A. The aryl hydrocarbon receptor in barrier organ physiology, immunology, and toxicology. Pharmacol Rev. (2015) 67:259–79. 10.1124/pr.114.009001 25657351

[3] Stockinger B Di Meglio P Gialitakis M Duarte JH. The aryl hydrocarbon receptor: multitasking in the immune system. Annu Rev Immunol. (2014) 32:403–32. 10.1146/annurev-immunol-032713-120245 24655296

[4] Quintana FJ Sherr DH. Aryl hydrocarbon receptor control of adaptive immunity. Pharmacol Rev. (2013) 65:1148–61. 10.1124/pr.113.007823 23908379 PMC3799235

[5] Wheeler MA Rothhammer V Quintana FJ. Control of immune-mediated pathology via the aryl hydrocarbon receptor. J Biol Chem. (2017) 292:12383–9. 10.1074/jbc.R116.767723 28615443 PMC5535014

[6] Shinde R Hezaveh K Halaby MJ Kloetgen A Chakravarthy A da Silva Medina T. Apoptotic cell-induced AhR activity is required for immunological tolerance and suppression of systemic lupus erythematosus in mice and humans. Nat Immunol. (2018) 19:571–82. 10.1038/s41590-018-0107-1 29760532 PMC5976527

[7] Wu D Li W Lok P Matsumura F Vogel CF. AhR deficiency impairs expression of LPS-induced inflammatory genes in mice. Biochem Biophys Res Commun. (2011) 410:358–63. 10.1016/j.bbrc.2011.06.018 21683686 PMC3137281

[8] Sekine H Mimura J Oshima M Okawa H Kanno J Igarashi K. Hypersensitivity of aryl hydrocarbon receptor-deficient mice to lipopolysaccharide-induced septic shock. Mol Cell Biol. (2009) 29:6391–400. 10.1128/MCB.00337-09 19822660 PMC2786870

[9] Bessede A Gargaro M Pallotta MT Matino D Servillo G Brunacci C. Aryl hydrocarbon receptor control of a disease tolerance defence pathway. Nature (2014) 511:184–90. 10.1038/nature13323 24930766 PMC4098076

[10] Yi YS. Caspase-11 non-canonical inflammasome: a critical sensor of intracellular lipopolysaccharide in macrophage-mediated inflammatory responses. Immunology (2017) 152:207–17. 10.1111/imm.12787 28695629 PMC5588777

[11] Na YR Jung D Gu GJ Jang AR Suh YH Seok SH. The early synthesis of p35 and activation of CDK5 in LPS-stimulated macrophages suppresses interleukin-10 production. Sci Signal. (2015) 8:ra121. 10.1126/scisignal.aab3156 26602020

[12] Hanieh H. Toward understanding the role of aryl hydrocarbon receptor in the immune system: current progress and future trends. Biomed Res Int. (2014) 2014:520763. 10.1155/2014/520763 24527450 PMC3914515

[13] Masuda K Kimura A Hanieh H Nguyen NT Nakahama T Chinen I. Aryl hydrocarbon receptor negatively regulates LPS-induced IL-6 production through suppression of histamine production in macrophages. Int Immunol. (2011) 23:637–45. 10.1093/intimm/dxr072 21930594

[14] Kimura A Naka T Nakahama T Chinen I Masuda K Nohara K. Aryl hydrocarbon receptor in combination with Stat1 regulates LPS-induced inflammatory responses. J Exp Med. (2009) 206:2027–35. 10.1084/jem.20090560 19703987 PMC2737163

[15] Huai WW Zhao R Song H Zhao J Zhang L Zhang LN. Aryl hydrocarbon receptor negatively regulates NLRP3 inflammasome activity by inhibiting NLRP3 transcription. Nat Commun. (2014) 5:5738. 10.1038/ncomms5738 25141024

[16] Gabrysova L Howes A Saraiva M O'Garra A. The regulation of IL-10 expression. Curr Top Microbiol Immunol. (2014) 380:157–90. 10.1007/978-3-662-43492-5_8 25004818

[17] Zahlten J Steinicke R Bertrams W Hocke AC Scharf S Schmeck B. TLR9- and Src-dependent expression of Krueppel-like factor 4 controls interleukin-10 expression in pneumonia. Eur Respir J. (2013) 41:384–91. 10.1183/09031936.00196311 22653776

[18] Kemp KL Levin SD Stein PL. Lck regulates IL-10 expression in memory-like Th1 cells. Eur J Immunol. (2010) 40:3210–9. 10.1002/eji.201040699 21061443 PMC3517127

[19] Norkina O Dolganiuc A Shapiro T Kodys K Mandrekar P Szabo G. Acute alcohol activates STAT3, AP-1, and Sp-1 transcription factors via the family of Src kinases to promote IL-10 production in human monocytes. J Leukoc Biol. (2007) 82:752–62. 10.1189/jlb.0207099 17575268

[20] Benkhart EM Siedlar M Wedel A Werner T Ziegler-Heitbrock HW. Role of Stat3 in lipopolysaccharide-induced IL-10 gene expression. J Immunol. (2000) 165:1612–7 10903771 10.4049/jimmunol.165.3.1612

[21] Li P Spolski R Liao W Wang L Murphy TL Murphy KM. BATF-JUN is critical for IRF4-mediated transcription in T cells. Nature (2012) 490:543–6. 10.1038/nature11530 22992523 PMC3537508

[22] Murray PJ. Understanding and exploiting the endogenous interleukin-10/STAT3-mediated anti-inflammatory response. Curr Opin Pharmacol. (2006) 6:379–86. 10.1016/j.coph.2006.01.010 16713356

[23] Staples KJ Smallie T Williams LM Foey A Burke B Foxwell BM. IL-10 induces IL-10 in primary human monocyte-derived macrophages via the transcription factor Stat3. J Immunol. (2007) 178:4779–85. 17404258 10.4049/jimmunol.178.8.4779

[24] Kaiser F Cook D Papoutsopoulou S Rajsbaum R Wu X Yang HT. TPL-2 negatively regulates interferon-beta production in macrophages and myeloid dendritic cells. J Exp Med. (2009) 206:1863–71. 10.1084/jem.20091059 19667062 PMC2737152

[25] Ananieva O Darragh J Johansen C Carr JM McIlrath J Park JM. The kinases MSK1 and MSK2 act as negative regulators of Toll-like receptor signaling. Nat Immunol. (2008) 9:1028–36. 10.1038/ni.1644 18690222

[26] Weichhart T Costantino G Poglitsch M Rosner M Zeyda M Stuhlmeier KM. The TSC-mTOR signaling pathway regulates the innate inflammatory response. Immunity (2008) 29:565–77. 10.1016/j.immuni.2008.08.012 18848473

[27] Busbee PB Rouse M Nagarkatti M Nagarkatti PS. Use of natural AhR ligands as potential therapeutic modalities against inflammatory disorders. Nutr Rev. (2013) 71:353–69. 10.1111/nure.12024 23731446 PMC4076843

[28] Tsai CF Hsieh TH Lee JN Hsu CY Wang YC Lai FJ. Benzyl butyl phthalate induces migration, invasion, and angiogenesis of Huh7 hepatocellular carcinoma cells through nongenomic AhR/G-protein signaling. BMC Cancer (2014) 14:556. 10.1186/1471-2407-14-556 25081364 PMC4131049

[29] Dong B Nishimura N Vogel CF Tohyama C Matsumura F. TCDD-induced cyclooxygenase-2 expression is mediated by the nongenomic pathway in mouse MMDD1 macula densa cells and kidneys. Biochem Pharmacol. (2010) 79:487–97. 10.1016/j.bcp.2009.08.031 19782052 PMC2796630

[30] Sciullo EM Dong B Vogel CF Matsumura F. Characterization of the pattern of the nongenomic signaling pathway through which TCDD-induces early inflammatory responses in U937 human macrophages. Chemosphere (2009) 74:1531–7. 10.1016/j.chemosphere.2008.11.010 19162293 PMC2879335

[31] Kimura A Naka T Nohara K Fujii-Kuriyama Y Kishimoto T. Aryl hydrocarbon receptor regulates Stat1 activation and participates in the development of Th17 cells. Proc Natl Acad Sci USA. (2008) 105:9721–6. 10.1073/pnas.0804231105 18607004 PMC2474493

[32] Nguyen NT Kimura A Nakahama T Chinen I Masuda K Nohara K. Aryl hydrocarbon receptor negatively regulates dendritic cell immunogenicity via a kynurenine-dependent mechanism. Proc Natl Acad Sci USA. (2010) 107:19961–6. 10.1073/pnas.1014465107 21041655 PMC2993339

[33] Vogel CFA Khan EM Leung PSC Gershwin ME Chang WLW Wu DL Cross-talk between aryl hydrocarbon receptor and the inflammatory response a role for nuclear factor-kappa B. J Biol Chem. (2014) 289:1866–75. 10.1074/jbc.M113.505578 24302727 PMC3894361

[34] Dong B Cheng W Li W Zheng J Wu D Matsumura F. FRET analysis of protein tyrosine kinase c-Src activation mediated via aryl hydrocarbon receptor. Biochim Biophys Acta (2011) 1810:427–31. 10.1016/j.bbagen.2010.11.007 21145940 PMC3049970

[35] Mezrich JD Fechner JH Zhang XJ Johnson BP Burlingham WJ Bradfield CA. An interaction between kynurenine and the aryl hydrocarbon receptor can generate regulatory T cells. J Immunol. (2010) 185:3190–8. 10.4049/jimmunol.0903670 20720200 PMC2952546

[36] Vondracek J Umannova L Machala M. Interactions of the aryl hydrocarbon receptor with inflammatory mediators: beyond CYP1A regulation. Curr Drug Metab. (2011) 12:89–103. 21401513 10.2174/138920011795016827

[37] Kerkvliet NI. AHR-mediated immunomodulation: the role of altered gene transcription. Biochem Pharmacol. (2009) 77:746–60. 10.1016/j.bcp.2008.11.021 19100241 PMC2662368

[38] Gandhi R Kumar D Burns EJ Nadeau M Dake B Laroni A. Activation of the aryl hydrocarbon receptor induces human type 1 regulatory T cell-like and Foxp3(+) regulatory T cells. Nat Immunol. (2010) 11:846–53. 10.1038/ni.1915 20676092 PMC2929008

[39] Apetoh L Quintana FJ Pot C Joller N Xiao S Kumar D. The aryl hydrocarbon receptor interacts with c-Maf to promote the differentiation of type 1 regulatory T cells induced by IL-27. Nat Immunol. (2010) 11:854–61. 10.1038/ni.1912 20676095 PMC2940320

[40] Mariuzzi L Domenis R Orsaria M Marzinotto S Londero AP Bulfoni M. Functional expression of aryl hydrocarbon receptor on mast cells populating human endometriotic tissues. Lab Invest. (2016) 96:959–71. 10.1038/labinvest.2016.74 27348627 PMC5008463

[41] Wagage S John B Krock BL Hall AO Randall LM Karp CL. The aryl hydrocarbon receptor promotes IL-10 production by NK cells. J Immunol. (2014) 192:1661–70. 10.4049/jimmunol.1300497 24403534 PMC3955958

[42] Lee JA Hwang JA Sung HN Jeon CH Gill BC Youn HJ. 2,3,7,8-Tetrachlorodibenzo-p-dioxin modulates functional differentiation of mouse bone marrow-derived dendritic cells Downregulation of RelB by 2,3,7,8-tetrachlorodibenzo-p-dioxin. Toxicol Lett. (2007) 173:31–40. 10.1016/j.toxlet.2007.06.012 17681673

[43] Vogel CF Wu D Goth SR Baek J Lollies A Domhardt R. Aryl hydrocarbon receptor signaling regulates NF-kappaB RelB activation during dendritic-cell differentiation. Immunol Cell Biol. (2013) 91:568–75. 10.1038/icb.2013.43 23999131 PMC3806313

[44] Climaco-Arvizu S Dominguez-Acosta O Cabanas-Cortes MA Rodriguez-Sosa M Gonzalez FJ Vega L. Aryl hydrocarbon receptor influences nitric oxide and arginine production and alters M1/M2 macrophage polarization. Life Sci. (2016) 155:76–84. 10.1016/j.lfs.2016.05.001 27153778 PMC6300993

[45] Tsai MJ Wang TN Lin YS Kuo PL Hsu YL Huang MS. Aryl hydrocarbon receptor agonists upregulate VEGF secretion from bronchial epithelial cells. J Mol Med. (2015) 93:1257–69. 10.1007/s00109-015-1304-0 26076680

[46] Ng HY Yisireyili M Saito S Lee CT Adelibieke Y Nishijima F. Indoxyl sulfate downregulates expression of Mas receptor via OAT3/AhR/Stat3 pathway in proximal tubular cells. PLoS ONE (2014) 9:e91517. 10.1371/journal.pone.0091517 24614509 PMC3948887

[47] Takanaga H Yoshitake T Yatabe E Hara S Kunimoto M. Beta-naphthoflavone disturbs astrocytic differentiation of C6 glioma cells by inhibiting autocrine interleukin-6. J Neurochem. (2004) 90:750–7. 10.1111/j.1471-4159.2004.02681.x 15255954

[48] Liu X Hu H Fan H Zuo D Shou Z Liao Y. The role of STAT3 and AhR in the differentiation of CD4+ T cells into Th17 and Treg cells. Medicine (2017) 96:e6615. 10.1097/MD.0000000000006615 28445259 PMC5413224

[49] Yeste A Mascanfroni ID Nadeau M Burns EJ Tukpah AM Santiago A. IL-21 induces IL-22 production in CD4+ T cells. Nat Commun. (2014) 5:3753. 10.1038/ncomms4753 24796415 PMC4157605

[50] Chiappini F Baston JI Vaccarezza A Singla JJ Pontillo C Miret N. Enhanced cyclooxygenase-2 expression levels and metalloproteinase 2 and 9 activation by Hexachlorobenzene in human endometrial stromal cells. Biochem Pharmacol. (2016) 109:91–104. 10.1016/j.bcp.2016.03.024 27038655

[51] Xie G Peng Z Raufman JP. Src-mediated aryl hydrocarbon and epidermal growth factor receptor cross talk stimulates colon cancer cell proliferation. Am J Physiol Gastrointest Liver Physiol. (2012) 302:G1006–15. 10.1152/ajpgi.00427.2011 22361730 PMC3362076

[52] Rey-Barroso J Colo GP Alvarez-Barrientos A Redondo-Munoz J Carvajal-Gonzalez JM Mulero-Navarro S. The dioxin receptor controls beta1 integrin activation in fibroblasts through a Cbp-Csk-Src pathway. Cell Signal. (2013) 25:848–59. 10.1016/j.cellsig.2013.01.010 23333462

[53] Bock KW Kohle C. Ah receptor: dioxin-mediated toxic responses as hints to deregulated physiologic functions. Biochem Pharmacol. (2006) 72:393–404. 10.1016/j.bcp.2006.01.017 16545780

[54] Park J Kim S Joh J Remick SC Miller DM Yan J. MLLT11/AF1q boosts oncogenic STAT3 activity through Src-PDGFR tyrosine kinase signaling. Oncotarget (2016) 7:43960–73. 10.18632/oncotarget.9759 27259262 PMC5190071

[55] Li R Yanjiao G Wubin H Yue W Jianhua H Huachuan Z. Secreted GRP78 activates EGFR-SRC-STAT3 signaling and confers the resistance to sorafeinib in HCC cells. Oncotarget (2017) 8:19354–64. 10.18632/oncotarget.15223 28423613 PMC5386689

