# Supplementary figures and images for: Aryl Hydrocarbon Receptor Promotes IL-10 Expression in Inflammatory Macrophages Through Src-STAT3 Signaling Pathway
Source: Front Immunol. 2018 Sep 19;9:2033. doi: 10.3389/fimmu.2018.02033 (PMC6156150; doi:10.3389/fimmu.2018.02033)

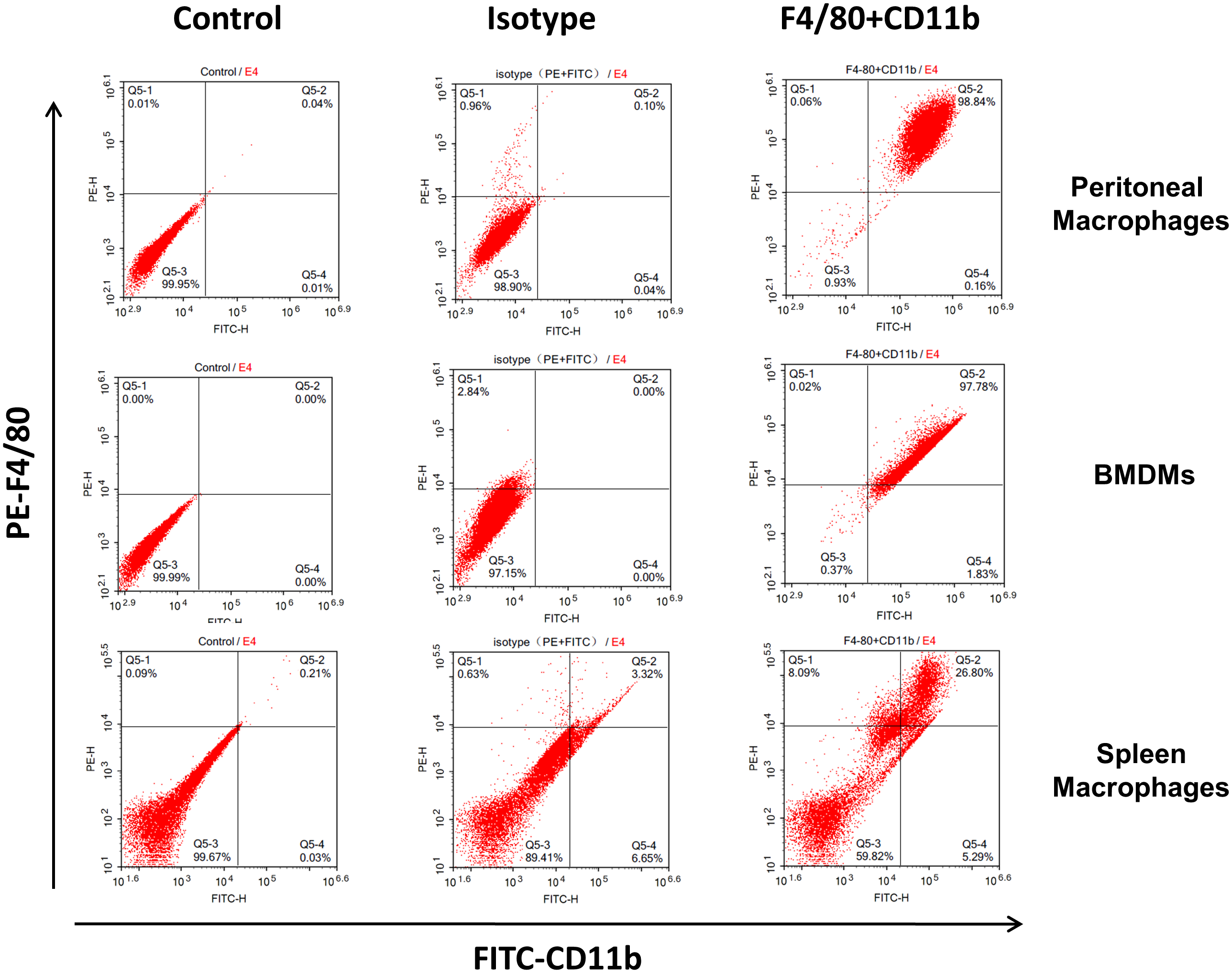

Supplement: Supplementary file 1 [file Image_1.TIF]

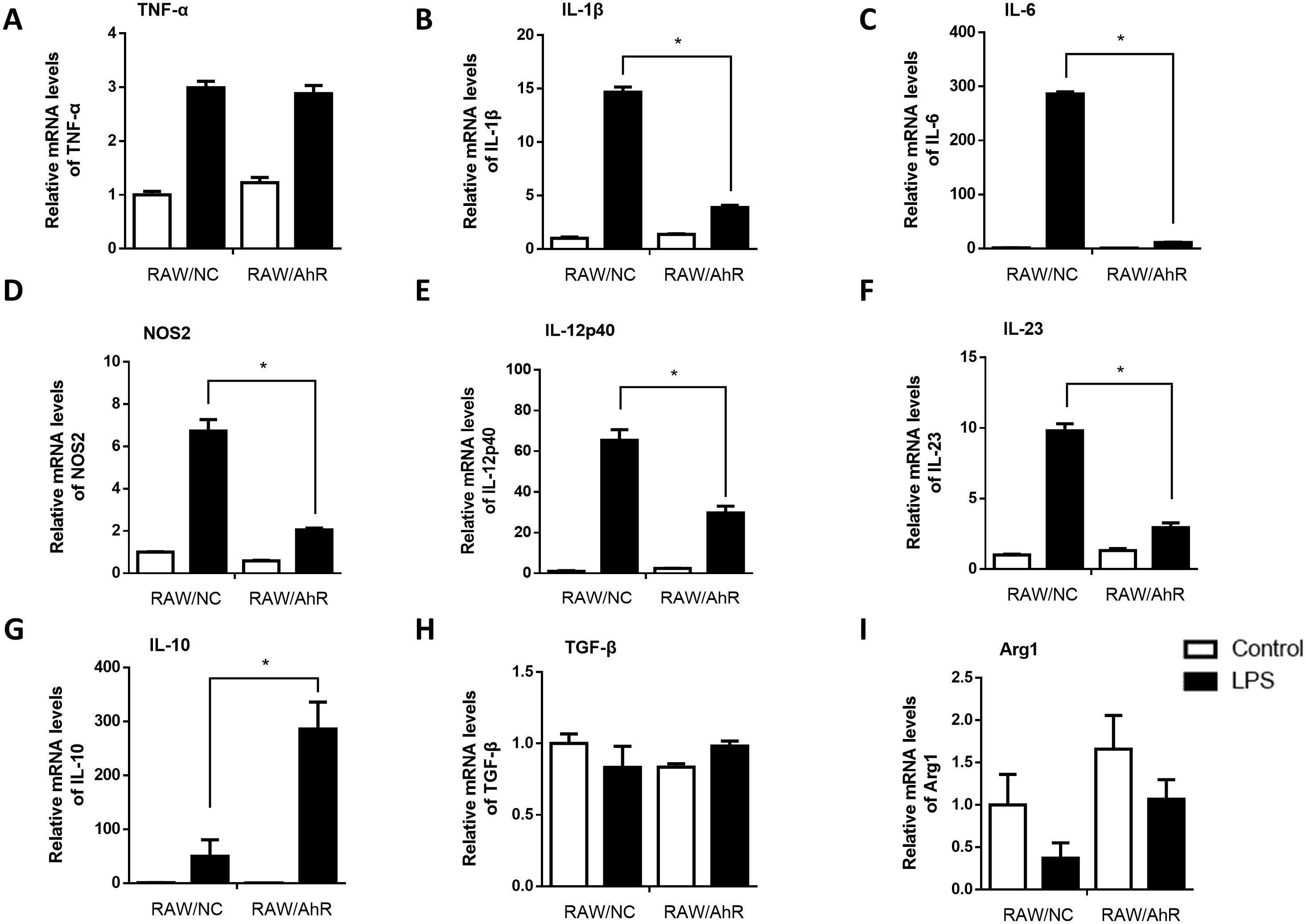

Supplement: Supplementary file 2 [file Image_2.TIF]

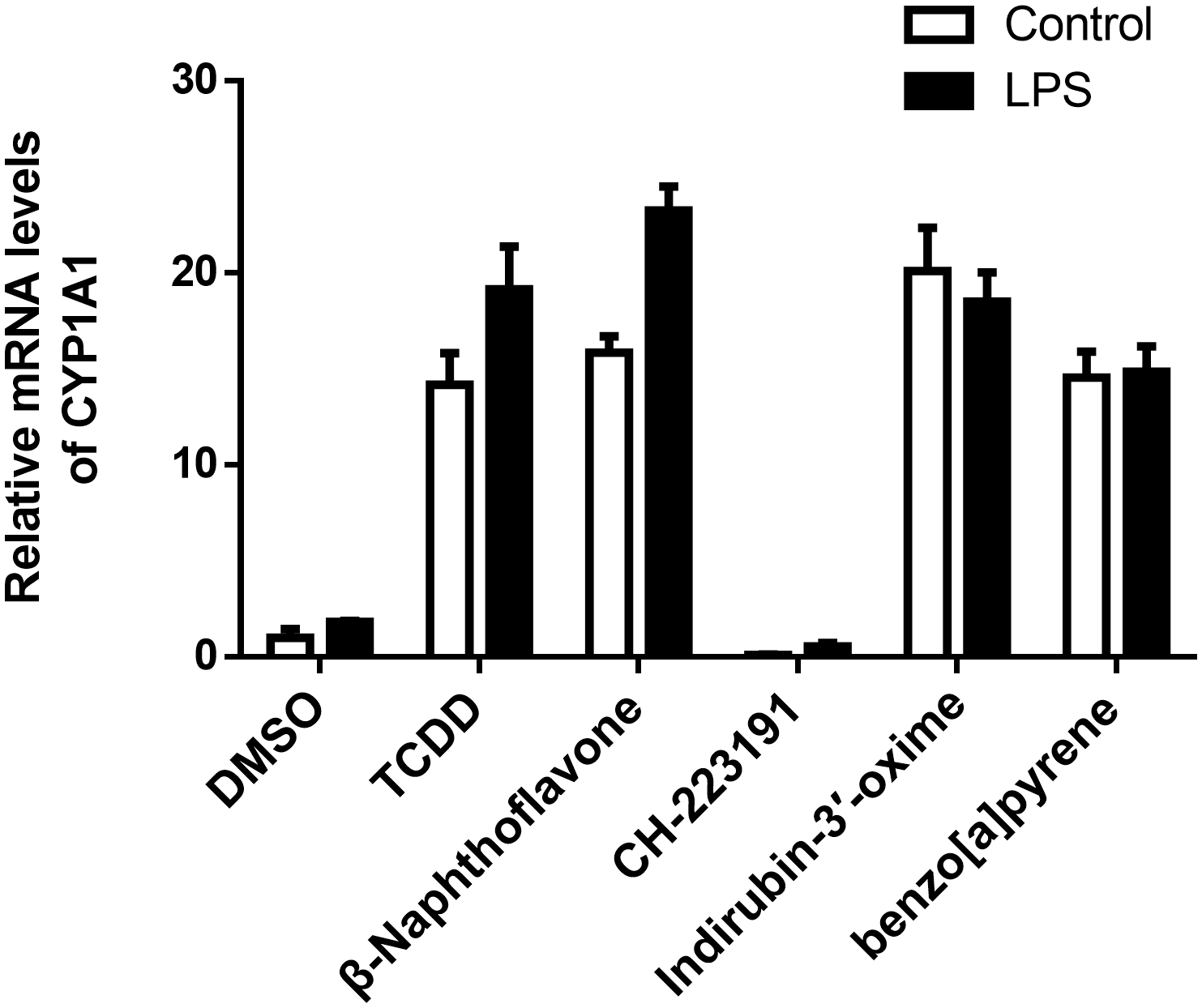

Supplement: Supplementary file 3 [file Image_3.TIF]

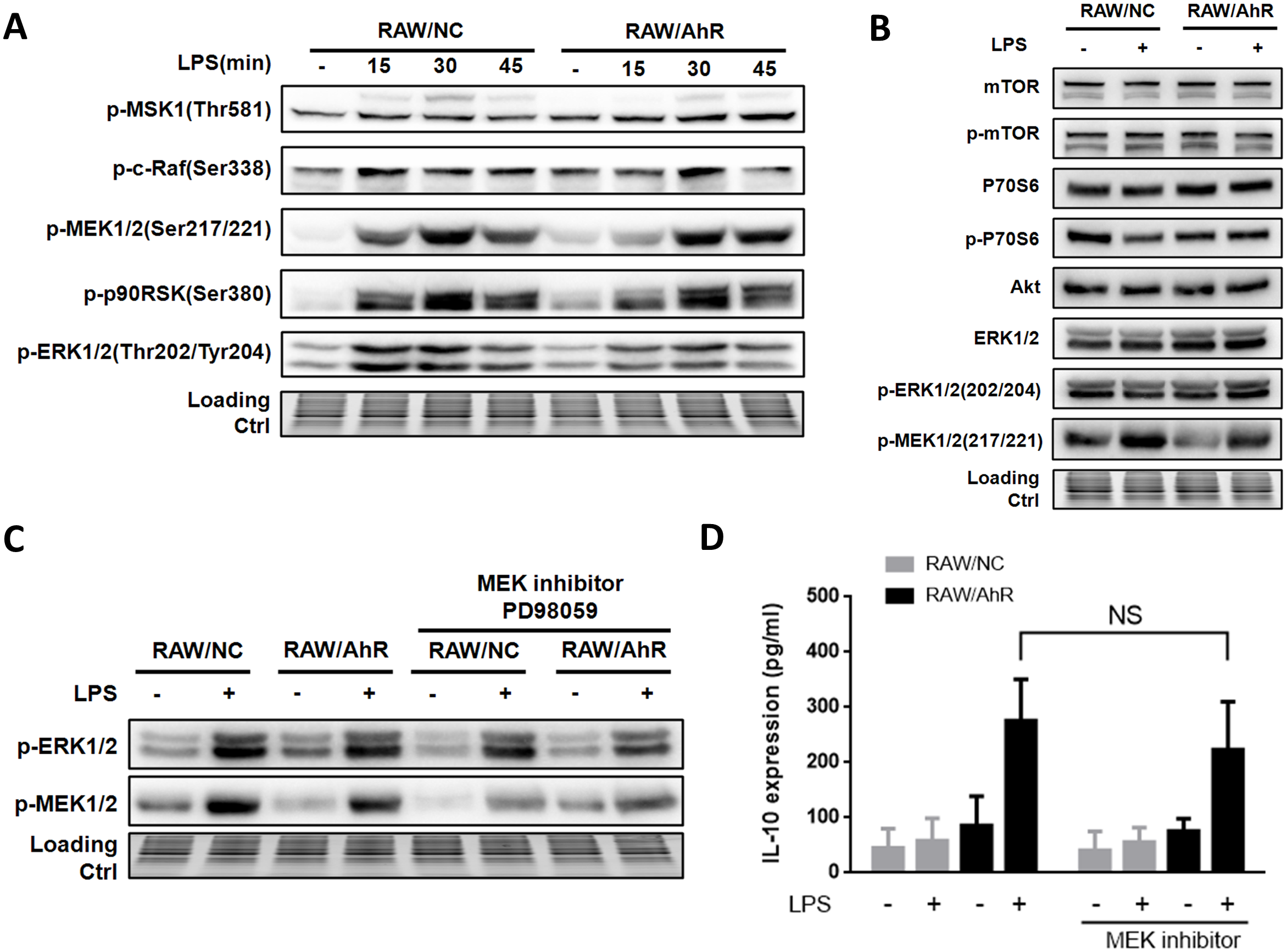

Supplement: Supplementary file 4 [file Image_4.TIF]

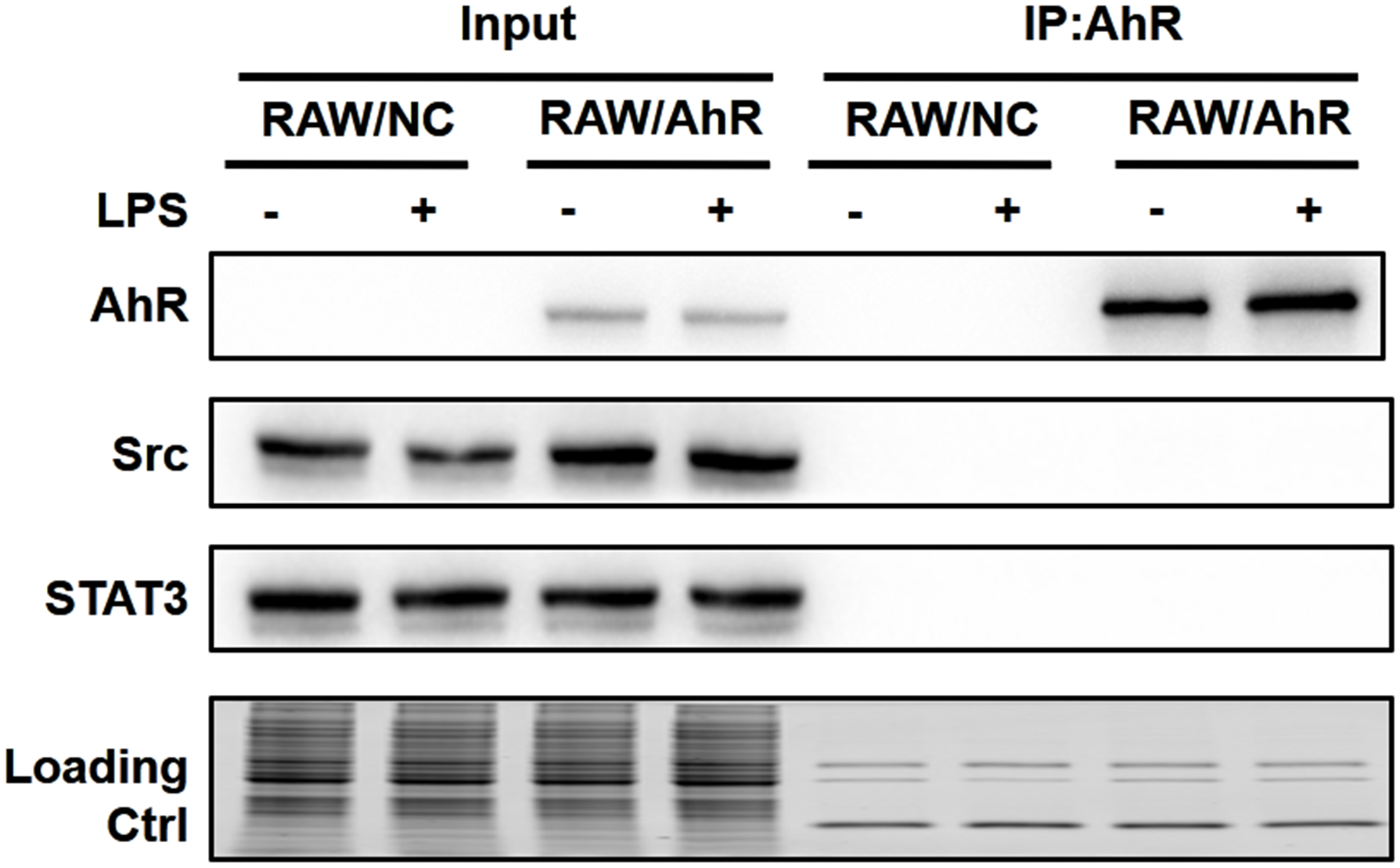

Supplement: Supplementary file 5 [file Image_5.TIF]
